# Neurofeedback Training Facilitates Awareness and Enhances Emotional Well-being Associated with Real-World Meditation Practice: A 7-T MRI Study

**DOI:** 10.1007/s12671-025-02671-z

**Published:** 2025-09-26

**Authors:** Saampras Ganesan, Nicholas T. Van Dam, Sunjeev K. Kamboj, Aki Tsuchiyagaito, Matthew D. Sacchet, Masaya Misaki, Bradford A. Moffat, Valentina Lorenzetti, Andrew Zalesky

**Affiliations:** 1 Department of Biomedical Engineering, The University of Melbourne, Melbourne, Australia; 2 Department of Psychiatry, The University of Melbourne, Melbourne, Australia; 3 Contemplative Studies Centre, Melbourne School of Psychological Sciences, The University of Melbourne, Melbourne, Australia; 4 Research Department of Clinical, Educational and Health Psychology, University College London, London, United Kingdom; 5 Neuroscience of Addiction and Mental Health Program, Healthy Brain and Mind Research Centre, School of Behavioral and Health Sciences, Faculty of Health, Australian Catholic University, Melbourne, Australia; 6 Melbourne Brain Centre Imaging Unit, Department of Radiology, The University of Melbourne, Melbourne, Australia; 7 Laureate Institute for Brain Research, Tulsa, OK USA; 8 Meditation Research Program, Department of Psychiatry, Massachusetts General Hospital, Harvard Medical School, Boston, Massachusetts, USA; 9 The University of Tulsa, Oxley College of Health & Natural Sciences, Tulsa, OK USA

**Keywords:** Meditation, Neurofeedback, 7-T fMRI, Emotional well-being, Mindful awareness, Posterior cingulate cortex, Dorsolateral prefrontal cortex

## Abstract

**Objectives:**

Novice meditators often struggle to recognise and intentionally disengage from self-referential thought during meditation. We investigated whether personalised high-precision neurofeedback (NF) training improves volitional disengagement from self-referential thought during meditation and enhances meditation’s outcomes.

**Method:**

In a single-blind, controlled study, novices received 2 days of veridical (*n* = 20) or sham (*n* = 20) 7-T fMRI NF targeting posterior cingulate cortex (PCC) deactivation during meditation. After NF training, at-home meditation practice was monitored for 1 week, followed by an in-lab behavioural assessment.

**Results:**

Both groups reported similar perceptions of NF contingency, performance, and expectancy (*p* > 0.05), suggesting effective participant blinding. PCC deactivation during NF-guided meditation was comparable across groups (*p* > 0.05). Veridical NF group showed significantly stronger negative functional coupling (*d* = 0.59) between PCC and dorsolateral prefrontal cortex (DLPFC), significantly greater mindful awareness (*d* = 0.41) and emotional well-being (*d* = 0.40) associated with 1-week practice, and significant correlation (*r* = 0.71, *p* < 0.01) between emotional well-being and PCC-DLPFC negative coupling.

**Conclusions:**

These findings suggest that high-precision NF can improve novices’ ability to volitionally disengage from self-referential thought during meditation, thereby fostering greater mindful awareness in real-world practice and promoting emotional well-being.

**Preregistration:**

This exploratory study was not preregistered.

**Supplementary Information:**

The online version contains supplementary material available at 10.1007/s12671-025-02671-z.

Meditation is a widespread contemplative practice that involves training attention and cultivating active and receptive awareness of thoughts, emotions, sensations, and mind–body perceptions (Sparby & Sacchet, 2021). Meditation enhances emotion regulation, weakens maladaptive psychological patterns, imparts beneficial neural changes, improves mindful awareness, and promotes overall well-being, offering transdiagnostic neuropsychological benefits in alleviating pervasive mental health and mood disorders (Bowles et al., 2022; Galante et al., 2021; Ganesan et al., 2024; Greeson et al., 2018).

Among various meditation practices, a widely practised and studied meditation technique, i.e. focused attention meditation, typically instructs one to focus on bodily breathing sensations, recognise and disengage from mental distractions when they arise, and refocus on breathing (Lutz et al., 2008). Focused attention meditation forms an integral component of mindfulness practice (Hölzel et al., 2011). Developing attentional skills through focused attention meditation is crucial for progressing in mindfulness and other meditation practices (Ganesan et al., 2022, 2023; Lutz et al., 2015; Sparby & Sacchet, 2024). Specifically, across diverse meditation practices, such as open-monitoring (Hölzel et al., 2011; Lutz et al., 2008), loving-kindness and compassion (Buddharakkhita, 2020; Dahl et al., 2015), and transcendental and stillness meditation (Woods et al., 2023), the core process involves repeatedly recognising and overcoming mental distractions to redirect attention onto the intended goal. In focused attention meditation, this fundamental skill is most explicitly developed using the breath, a commonly employed tangible and controllable anchor that supports voluntary control and conscious observation (Jha et al., 2007; Laukkonen & Slagter, 2021).

However, novices are often deeply entrenched in self-referential thinking (Killingsworth & Gilbert, 2010; Laukkonen & Slagter, 2021; Zanesco et al., 2025) with limited meta-cognitive awareness and attention regulation capability (McVay et al., 2009), which can contribute to limited recognition of and intentional disengagement from mental distractions during meditation (Hölzel et al., 2011; Schooler et al., 2011). These factors can diminish the quality and psychological benefits of meditation practice, which may discourage continued practice (Brewer et al., 2013; Goldberg et al., 2020; Russ et al., 2017; Strohmaier & Goldberg, 2024). Currently, hundreds of hours of self-guided practice may be necessary to achieve significant relief from psychological distress (Bowles et al., 2022). Furthermore, the subjective/abstract nature of meditation and its instructions can complicate the monitoring of meditation practice (Galante et al., 2023; van Lutterveld et al., 2017).

Integrating technology like neurofeedback (NF) with meditation training can potentially contribute to addressing some of the challenges experienced in meditation by novice meditators. NF is an approach that delivers real-time feedback on personalised brain activity that can assist trainees in learning to self-regulate desired mental states, behaviours, or pathologies (Sitaram et al., 2017) (Fig. 1).

**Fig. 1:**
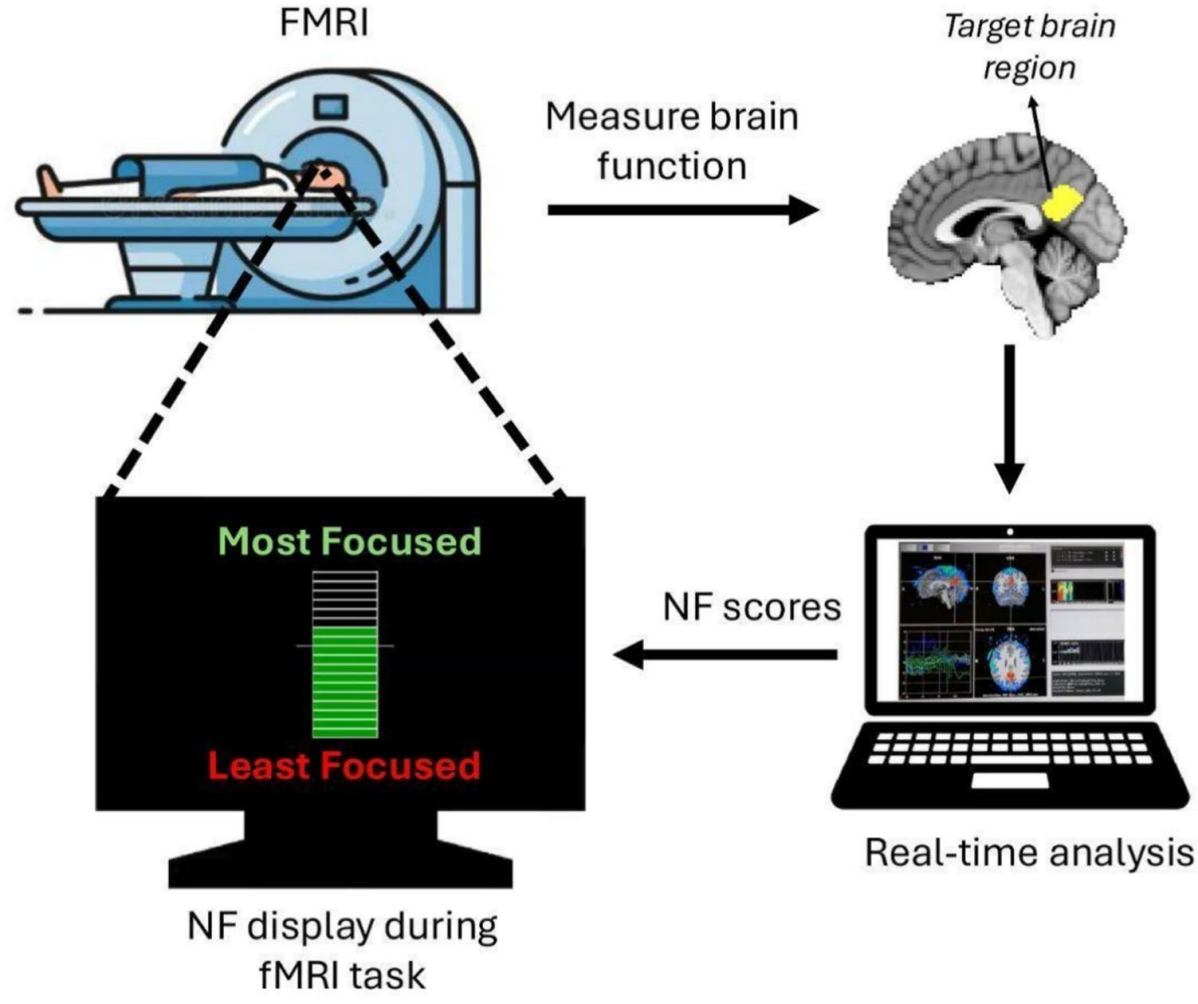
Schematic of real-time functional MRI (fMRI) neurofeedback (NF). The participant’s brain function is measured and analysed in real-time using fMRI, generating NF scores that are displayed to the participant (via a thermometer with incremental bars). The display facilitates self-regulation of the target brain function in a closed-loop. In the current study, the posterior cingulate cortex (PCC), shown in yellow, is used as the target brain region

Emerging evidence suggests that NF training leverages reinforcement learning (Lubianiker et al., 2022), with capacity to enduringly impact behaviour and brain function in healthy individuals, while also aiding in the normalisation of brain function in various pathologies (Krause et al., 2024; Rance et al., 2018; Sitaram et al., 2017). While NF using electroencephalography (EEG) is more common and cost-effective, NF using functional magnetic resonance imaging (fMRI) offers comparatively superior neuroanatomical resolution, with capacity for more significant positive effects on neural, clinical, and behavioural measures after shorter training durations (Dudek & Dodell-Feder, 2021; Krause et al., 2024; Sitaram et al., 2017; Thibault & Raz, 2017; Thibault et al., 2018). For example, a single fMRI-NF session significantly improved attention in healthy participants compared to control groups receiving sham NF, no NF, and non-neural NF (deBettencourt et al., 2015). Similarly, a randomised controlled trial (RCT) showed that two fMRI-NF sessions helped patients with depression upregulate amygdala activity, significantly reducing symptoms compared to a sham-NF group (Young et al., 2017).

FMRI-NF also offers a unique opportunity to explore causal relationships between brain activity, behaviour, and pathologies. Specifically, it enables endogenous manipulation of brain activity/state as an independent variable, while bypassing conscious awareness and providing comprehensive non-invasive neuroanatomical coverage (Iordan et al., 2024; Sitaram et al., 2017; Sulzer et al., 2013; Taschereau-Dumouchel et al., 2018). The advent of ultra-high field 7-T MRI, offering superior neuroanatomical precision, statistical power, and signal quality compared to 3-T MRI, holds promise for further enhancing the neuroanatomical precision achievable with fMRI-NF (Pohmann et al., 2016; Torrisi et al., 2018).

Real-time, precise, and objective feedback from the brain during meditation can provide external scaffolding for meta-awareness, potentially supporting beginners in recognising distractions and gaining better control over attention (Brandmeyer & Reggente, 2023; Treves et al., 2024). Strengthening of meta-awareness and attentional control can subsequently improve the ability of beginning meditators to disengage from distractions, facilitating deeper insights into mind–body phenomena. Importantly, following NF-guided meditation training, individuals can continue developing their NF-acquired meditation skills independently through real-world practice, without frequent reliance on the specialised NF equipment (Krause et al., 2024). Moreover, combining NF with meditation offers a unique opportunity to investigate the neural mechanisms underlying meditative states, potentially identifying causal links between brain activity and meditation outcomes (Treves et al., 2024).

Key targets for fMRI-NF with meditation have been the posterior cingulate cortex (PCC) and the broader default-mode network (DMN) (Treves et al., 2024). Particularly, the PCC, within the DMN, has been widely implicated in mental activity and self-referential thought (Leech & Sharp, 2014). The PCC is often found to reliably reduce its activation (i.e. deactivate) during focused attention meditation relative to a control condition, as indicated by systematic syntheses of the fMRI literature on focused attention meditation (Ganesan et al., 2022) as well as neurophenomenological evidence from expert meditators (Garrison et al., 2013). Notably, a recently proposed neurocognitive model of focused attention meditation (Ganesan et al., 2022), derived from the systematic syntheses of fMRI literature, suggests that deactivation of the DMN—linked to self-referential thought—is typically accompanied by activation of the salience network (SN) and central executive network (CEN), which support interoceptive awareness and cognitive control, respectively. This interplay is thought to facilitate the volitional reduction of self-referential thought, enhancing focus and awareness of the breath.

While fMRI-NF with meditation has demonstrated preliminary efficacy in modulating the PCC and DMN (Bauer et al., 2020; Garrison et al., 2013; Kim et al., 2019; Kirlic et al., 2022; Pamplona et al., 2020; Zhang et al., 2023), its impact on real-world, self-guided meditation practice and associated psychological benefits remains unclear (Treves et al., 2024). Specifically, although NF has the potential to facilitate transfer of learned skills to real-world environments without the need for ongoing feedback or NF equipment (Krause et al., 2024), it is currently unclear whether meditation NF can enhance the capacity of trainees to deepen their meditation practice in everyday settings.

Most fMRI meditation NF studies to date have not included a control group to rule out placebo or non-specific effects (Treves et al., 2024). Among the few that did, Bauer et al. (2020) incorporated a within-subject control NF task conducted 12 weeks after the original NF training with a different NF target, potentially introducing confounds such as differences in strategy, expectancy, or feedback controllability. Pamplona et al. (2023) included a control group that received no MRI or NF exposure, thereby not accounting for NF- and MRI-specific confounds. While H. C. Kim et al. (2019) implemented a well-matched control group, they found no significant between-group differences in outcomes. Consequently, the effectiveness of fMRI meditation NF beyond placebo remains uncertain (Treves et al., 2024).

Although intermittent feedback delivery may improve NF learning by allowing participants to focus on the task without distractions from continuously updated feedback or fMRI haemodynamic delays (Lubianiker et al., 2019; Paret et al., 2019; Stoeckel et al., 2014), only one study (Pamplona et al., 2023) explored this approach. All others relied on continuously updated feedback (NF score updated every fMRI volume). Furthermore, no study has examined whether fMRI meditation NF influences real-world meditation practice or associated mental health outcomes, raising concerns about the translational utility of fMRI meditation NF outside the lab. Similarly, meditation NF leveraging 7-T fMRI remains unexplored. Finally, only Kirlic et al. (2022) adhered to the CRED-NF checklist (Ros et al., 2020) that was designed for fMRI-NF methodological transparency.

To address these critical limitations, we conducted a controlled, single-blind, longitudinal, proof-of-concept study to assess the impact of multi-day fMRI meditation NF training on mindful awareness (Tanay & Bernstein, 2013) during subsequent real-world meditation practice (i.e. self-guided silent meditation with eyes closed in a desired posture) among healthy novices. We hypothesised that the fMRI NF training, compared to control, will improve *intentional* engagement of the targeted brain region and produce greater improvements in mindful awareness during subsequent self-guided real-world meditation practice. We also tested the effect of post-NF real-world practice on emotional distress (a measure of negative emotional states related to depression, anxiety, and stress) (Lovibond & Lovibond, 1996) and on an objective proxy for mindfulness and attention (breath counting performance) (Levinson et al., 2014), anticipating that emotional distress will be lower and breath counting performance will be better compared to control. Overall, this study establishes a foundation for developing precise brain-based meditation training technology aimed at reducing the challenges and enhancing the effectiveness of real-world meditation practice in modern society.

## Method

### Participants

In total, 51 healthy, adult volunteers were found eligible and willing to participate, of which 11 did not complete the study requirements for the following reasons: withdrawal after the baseline session (*n* = 2), withdrawal during MRI scanning due to discomfort (*n* = 7), and cancellations caused by MRI scanner malfunctioning (*n* = 2). The final sample consisted of 40 participants (Online Resource ESM_1.pdf, Figure S1 for CONSORT schematic).

The participants were assigned to either an experimental group (*n* = 20), which meditated with real NF from their own brain activity, or a control group (*n* = 20), which meditated with sham NF derived from the brain activity of an experimental participant matched based on self-reported meditation experience (i.e. yoked-sham NF). The participants were not informed about the existence of a control group in the study (i.e. they did not have any knowledge about the existence of multiple groups) and were consequently blinded to their group assignment. Every participant received expert guidance and instruction on meditation technique, and a 3D printed model of their high-resolution anatomical brain image as a reward for volunteering.

The inclusion criteria were as follows: age between 19 and 50 years; self-reported interest in learning meditation; fluency in English; beginner at meditation — broadly defined as having irregular, sparse or no meditation practice, no meditation retreat experience over the past 2 years, cumulative lifetime meditation experience under 500 h and lifetime participation in less than two meditation retreats; and completed two prescribed audio-guided meditation sessions prior to baseline assessments. The exclusion criteria were self-reported lifetime clinical diagnoses of any neuropsychiatric (e.g. psychosis, addictions, depression, anxiety) or neurological (e.g. traumatic brain injury, epilepsy) disorders; lifetime consumption of any psychoactive medication (e.g. antidepressants, benzodiazepines, anti-psychotics) with or without prescription; regular or recent consumption of psychoactive (e.g. cannabis, psilocybin) drugs; alcohol-use disorder with a score of $\ge$ 4 for males and $\ge$ 3 for females in the Alcohol Use Disorders Identification Test (AUDIT-C) (Bush et al., 1998); or contraindications to MRI scanning. The study protocol components are reported in the CRED-nf checklist (Online Resource ESM_1.pdf, Table S1).

### Procedure

**Fig. 2:**
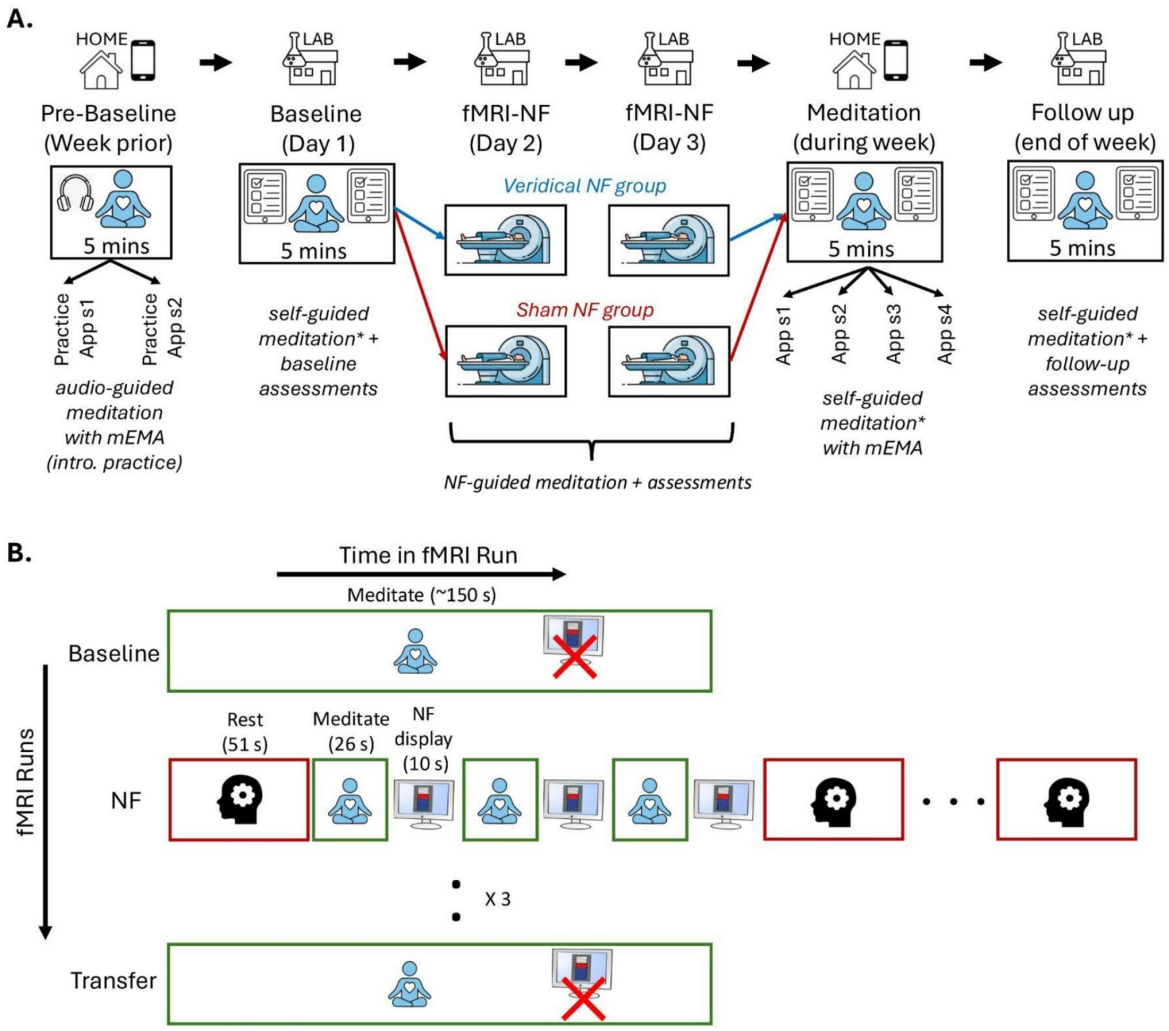
Schematic of the experimental designs used in this study. **A** The study included a pre-baseline period with two introductory 5-min audio-guided meditation sessions at home using the mEMA app, a baseline session including the first 5-minself-guided meditation on day 1, fMRI-neurofeedback (NF) training on days 2 and 3, a week of self-guided meditation (four 5-min sessions) at home using the mEMA app, and a follow-up session at the end of the week, which included the final 5-min self-guided meditation session. A thermometer with incremental bars was used to display real-time visual feedback during the NF sessions. **B** The fMRI design diagram outlines the NF training procedure, detailing fMRI runs (y-axis) and the conditions/tasks within each run (x-axis). Participants completed a baseline meditation run without NF, three NF runs involving rest (3 blocks) and meditation (6 blocks) with intermittent NF display, and a transfer meditation run without NF to evaluate transfer learning. In each NF run, there were three pairs of meditation and NF blocks between each pair of rest blocks. The same procedure was followed on both NF sessions (days 2 and 3). mEMA, mobile Ecological Momentary Assessment; NF, neurofeedback; sx, session x. *Immediately after each 5-min meditation session, participants completed the self-report State Mindfulness Scale (SMS) which assessed mindful awareness *during *the session, with pre-session SMS scores serving as a control for baseline mindful awareness

The study included several stages: non-MRI baseline assessments and a self-guided meditation session, two consecutive days of 7-T fMRI NF-guided meditation training, four self-guided meditation sessions at home during the following week, and non-MRI follow-up assessments with a self-guided meditation session after 1 week. The complete study design is illustrated in Fig. 2, with more details in Online Resource ESM_1.pdf, SM2.1-SM2.5.

During the week before baseline, participants completed two 5-min audio-guided focused attention meditation sessions using the mEMA app at home for familiarisation. At baseline in the lab, dispositional mindfulness (Five Facet Mindfulness Questionnaire (FFMQ) (Baer et al., 2006)), dispositional mind-wandering (Mind-Wandering Questionnaire (MWQ) (Mrazek et al., 2013)), dispositional anxiety (State and Trait Anxiety Inventory, Trait Module (STAI-T) (Spielberger et al., 1971)), and past-month sleep quality (Pittsburgh Sleep Quality Index (PSQI) (Buysse et al., 1989)) were measured to evaluate any relevant pre-existing differences between groups before the study commenced.

Other than that, participants completed a total of six 5-min sessions of self-guided, silent, eyes-closed focused attention meditation (on breathing sensations): one at baseline in the lab, four at home post-NF training via the mEMA app, and one at the 1-week follow-up in the lab (detailed meditation instructions can be found in Online Resource ESM_1.pdf, SM2). State Mindfulness Scale (SMS; Mind and Body subscales) (Tanay & Bernstein, 2013) and Stanford Sleepiness Scale (SSS) (Hoddes et al., 1973) were measured before and after each meditation session (six timepoints). Pre-meditation SMS assessed mindful awareness of thought and bodily experiences during 5 min before meditation, while post-meditation SMS assessed mindful awareness during the 5-min meditation period. SSS ratings, measuring momentary sleepiness, were used during analyses to control for the impact of drowsiness on meditation states and awareness.

The Depression Anxiety Stress Scales (DASS-21) (Lovibond & Lovibond, 1996) was administered at baseline and 1-week follow-up to measure negative emotional states (related to depression, stress, and anxiety), reflecting emotional distress during the week prior to baseline and the post-NF week of self-guided meditation. The Breath Counting Task (BCT) (Levinson et al., 2014) was assessed at baseline and follow-up, measuring task (% of correct breath count cycles) and probe (% of correct breath counts reported when probed) accuracy.

MRI data were acquired on a 7-T MRI scanner (Siemens Magnetom 7 T plus) using an 8/32 PTX/RX channel head coil. High-resolution whole-brain T1-weighted (T1w) anatomical images (3D-MP2RAGE; 0.75-mm isometric voxel size; TE/TR = 2 ms/5000 ms) and functional images (1.6-mm isometric voxel size; TE/TR = 22 ms/800 ms; multiband acceleration = 6) were acquired using multiband gradient-echo echo-planar imaging (EPI) sequence (see Online Resource ESM_1.pdf, SM2.6 for complete details). Concurrent physiological measurements were acquired using an MRI-compatible respiration belt and a pulse oximetry sensor, with data from the latter excluded due to faulty recordings. Real-time fMRI was implemented through Transmission Control Protocol (TCP) connection between the MRI console computer, the computer with real-time fMRI analysis software (Turbo-BrainVoyager v4.2), and the visual stimulation computer (with Psychtoolbox v3.1). Complete details are provided in Online Resource ESM_1.pdf, SM2.7.1.

The fMRI NF-guided meditation training was conducted over two consecutive days for each participant. Each fMRI session (Fig. 2B) comprised a baseline meditation task (2.5 min) without NF, three NF-guided meditation runs (10 min each), and a transfer meditation task (2.5 min) without NF. Each NF run had a blocked design: three blocks of rest (51 s each) and six blocks of focused attention meditation (26 s each). For the meditation condition, participants were instructed to maintain open eyes, focus on the sensations of natural breathing, and gently redirect their awareness to these sensations upon noticing distractions. For the rest condition, participants were instructed to breathe naturally and think freely with their eyes open. The MRI task instructions were adapted from (Ganesan et al., 2023) (see Online Resource ESM_1.pdf, SM2.8 for detailed instructions). NF scores were visually displayed (Online Resource ESM_1.pdf, Figure S2) after each meditation block. There were three pairs of meditation and NF blocks between each pair of rest blocks. After each fMRI run, SSS sleepiness ratings were acquired. After each fMRI session, participants rated the utility of NF, their meditation performance, and correspondence between NF scores and their meditative attention using 5-point Likert scales, and summarised their in-scanner meditation and rest strategies.

### Measures

FFMQ (Baer et al., 2006) was used to measure dispositional mindfulness, i.e. general tendency to be mindful in daily life. FFMQ shows high internal reliability (Cronbach’s *α* > 0.85) (Christopher et al., 2012) and comprises 39 self-report questions covering five mindfulness facets, i.e. observing, describing, acting with awareness, non-reactivity to inner experiences, and non-judging of inner experiences. Questions are scored from 1 to 5, with higher scores indicating a greater tendency towards mindfulness in daily life.

MWQ (Mrazek et al., 2013) was used to assess dispositional mind-wandering. It consists of six statements, each self-rated on a 6-point Likert scale. MWQ demonstrates high internal reliability (Cronbach’s *α* = 0.85) (Mrazek et al., 2013), and evaluates the extent of task-unrelated thoughts in daily activities, capturing both deliberate and spontaneous mind-wandering. Lower MWQ scores signify lower levels of dispositional mind-wandering, which are typically also related to higher mindful awareness in daily life (Mrazek et al., 2013).

STAI-T (Spielberger et al., 1971) was administered to measure dispositional anxiety. STAI-T comprises 20 self-report questions with 4-point Likert scale responses. Lower scores indicate lower levels of dispositional anxiety. STAI-T shows high internal reliability (Cronbach’s *α* > 0.85) and has been validated to assess anxiety in research and clinical settings (Barnes et al., 2002). Greater tendencies towards mindful qualities have been associated with lowered levels of dispositional anxiety (Fino et al., 2021).

PSQI (Buysse et al., 1989) was used to evaluate sleep quality of participants 1 month prior to study commencement. The PSQI is a validated and widely used clinical instrument with Cronbach’s *α* > 0.70 (Mollayeva et al., 2016). It comprises 19 individual items spanning seven components: subjective sleep quality, sleep latency, sleep duration, habitual sleep efficiency, sleep disturbances, use of sleeping medication, and daytime dysfunction. Lower total PSQI scores signify higher overall sleep quality over the past month. PSQI enabled identifying between-group differences in baseline sleep quality, which can potentially influence subsequent meditation and mindfulness states (Britton et al., 2014).

DASS-21 (Lovibond & Lovibond, 1996) was used to quantify emotional distress over a 1-week time interval. DASS-21 is a validated self-report questionnaire with high internal reliability (Cronbach’s *α* > 0.8) in non-clinical samples (Henry & Crawford, 2005). It comprises 21 questions with 4-point Likert rating responses, providing a quantitative measure of distress along the three axes of depression, anxiety, and stress over the past week. The total DASS-21 score (Cronbach’s *α* = 0.93) represents overall emotional distress, serving as a composite measure of negative emotional states related to depression, stress, and anxiety. Lower scores signify lesser distress.

The SMS (Tanay & Bernstein, 2013) is a self-report tool that measures perceived attention and mindful awareness of present-moment experiences over a specified period and context. When the context involves meditation, SMS responses capture mindful awareness during meditation. SMS comprises 21 statements with 5-point ratings divided into two subscales based on the object of mindful attention and awareness, i.e. SMS Body (Cronbach’s *α* > 0.80) for bodily sensations (six statements) and SMS Mind (Cronbach’s *α* > 0.90) for mental events (e.g. emotions, thought patterns; 15 statements). Higher scores indicate greater mindful awareness. SMS has consistently demonstrated excellent internal reliability (Cronbach’s *α* range = 0.80 to 0.95) (Ruimi et al., 2022). Crucially, SMS enables capturing dynamic changes in mindful awareness associated with meditation training, practice, and expertise (Ruimi et al., 2022).

The SSS, a widely used sleepiness measurement tool, consists of a single 8-point Likert scale where lower ratings indicate greater alertness. SSS ratings are used to control for the impact of drowsiness on meditation states and awareness.

The Breath Counting Task (BCT) (Levinson et al., 2014), an objective proxy of mindfulness skill, was used to evaluate attention to breathing cycles and their counts. In this task, participants count their breaths from 1 to 9 cyclically, registering each count with a button press. A left arrow button was pressed for the first eight counts, while a right arrow button was pressed for the ninth count of each cycle. At five pseudo-random instances during the task, participants were probed to verbally report their current breath count. Task accuracy is assessed by the percentage of correct count cycles, and probe accuracy is determined by the percentage of correctly reported counts when probed. The physiological accuracy of the breath counts was evaluated by correlating the count rates with the breathing rates recorded using a respiration belt (Vernier Science Education, OR, USA) worn during the task.

### Data Analyses

Behavioural data from two participants were excluded due to doubtful compliance with at-home app usage, resulting in *n* = 38 participants (19 per group) for behavioural analyses. The mEMA app usage compliance was assessed using automatically recorded start, end, and pause timestamps for each 5-min meditation recording— which included start and end bells separated by a 5-min period of silence. The excluded participants had multiple pause timestamps, with some sessions showing less than 5 min between start and end times, suggesting they may have skipped through some of the meditation periods without actual engagement.

Blinding and control were examined through non-parametric Wilcoxon rank sum tests, which assessed statistical differences in participants’ post-NF self-ratings (0–5 integer scores) from each NF session on (i) the utility of NF for learning meditation, (ii) in-scanner meditation performance, and (iii) the correspondence between NF scores and meditation. Pearson’s correlation was computed to evaluate the association between the sham and actual mean PCC signals associated with the control group in each NF session, using both online and offline denoised signals.

Group differences in all the assessments and characteristics measured at baseline—including age, sex, meditation experience (hours), dispositional mindfulness (FFMQ and sub-scales), dispositional mind-wandering (MWQ), dispositional anxiety (STAI-T), 1-month sleep quality (PSQI), 1-week emotional distress (DASS-21), and breath counting (BCT accuracy) — were evaluated using two-sample *t*-tests, except for sex and ethno-geographic background, where Chi-square tests were used to compare proportions between groups.

The correlation between each session’s mean offline denoised PCC beta values and mean online denoised PCC PSC was estimated to assess the validity of the PCC signals calculated online during NF sessions.

Analyses of covariance (ANCOVAs), within the general linear model (GLM) framework, were used to explore whether the online activation in the confound ROI, a proxy for online physiological fluctuations, differed significantly between groups during NF-guided meditation vs. rest. Specifically, this involved session-wise ANCOVAs to identify group differences in the confound ROI’s mean PSC during meditation relative to resting baseline, after adjusting for age and sex as covariates.

Primary behavioural analyses involved SMS, DASS-21, and BCT scores. To isolate the change in mindful awareness during real-world meditation practice, pre-meditation SMS scores, mean SSS ratings, age, and sex were regressed out from the post-meditation SMS scores of each 5-min meditation session. Repeated-measures ANCOVAs were performed on the resulting residuals from each participant across the six meditation sessions (timepoints), with time since baseline as the predictor variable. Each ANCOVA produced one regression slope per participant for each SMS subscale, which was subsequently entered into one-way analysis of variance (ANOVA) to examine group differences in slope values for each subscale separately (detailed description provided in Online Resource ESM_1.pdf, SM2.10).

Difference scores (follow-up minus baseline) for total DASS-21 were calculated, and ANCOVA was used to detect significant group differences in these difference scores, with age and gender as covariates. The strong correlations between the DASS-21 subscales at both timepoints (*r* (36) > 0.79, *p* < 0.0001) justified the use of total DASS-21 score instead of subscale scores for this analysis.

Difference scores (follow-up minus baseline) of BCT task performance and probe accuracies were calculated. ANCOVA was used to detect significant group differences in task accuracy difference scores, with age and gender as covariates. Group differences in probe accuracy difference scores were analysed using the non-parametric Wilcoxon rank-sum test, which is suitable for datasets with discrete values, such as integer scores ranging from 0 to 5. In addition to the two participants excluded from behavioural analyses (as aforementioned), one participant’s BCT data was not saved, resulting in *n* = 37 participants (*n* = 18 experimental, *n* = 19 control) for the BCT analysis.

False-discovery rate (FDR) correction at a level of 0.05 was applied to control across all the five behavioural tests (Online Resource ESM_1.pdf, Table S2). Effect sizes were estimated by transforming each resulting *t*-statistic to Cohen’s *d.*

Real-time fMRI preprocessing and analysis were conducted using Turbo-BrainVoyager (v4.2) and MATLAB, with visual cues and feedback managed by Psychtoolbox (v3.1). Online preprocessing steps included coregistration to anatomical and MNI space, motion correction, spatial smoothing, linear detrending, and physiological control through nuisance regression using percent signal change (PSC) from a confound region-of-interest (ROI) linked to physiological artefacts (see Online Resource ESM_1.pdf, SM2.7.2 for complete preprocessing details). The proxy confound ROI (shown in Online Resource ESM_1.pdf, Figure S4) encompassed midline anterior and posterior grey matter, white matter, and ventricular structures.

The target ROI for NF used in this study was the bilateral ventral PCC (Fig. 4A), defined using the Schaefer brain atlas (Schaefer et al., 2018) in standard MNI space. Notably, much of the midline DMN, including parts of the PCC outside bilateral ventral PCC, was incorporated into the confound ROI because these regions, adjacent to large vasculature, are particularly susceptible to respiration-induced blood flow changes (Birn et al., 2006). This susceptibility is further amplified when fMRI task conditions align closely with physiological response fluctuations (Birn et al., 2006, 2009), which is often true for breath-focused meditation (Ahani et al., 2013; Farb et al., 2013; Soni & Muniyandi, 2019; Weng et al., 2020). While the conservative confound region may have inadvertently removed meaningful variance associated with the target PCC region, priority was placed on maximising online control over physiological confounds and minimising the compounded impact of physiological noise on both fMRI NF and meditation-related fMRI signals.

The active condition involved focused attention meditation, while the control condition involved rest, with NF scores calculated intermittently after each active condition. Real-time voxel-wise PSC of the target PCC ROI was estimated through incremental GLM in Turbo-BrainVoyager. The mean PSC from each time point was estimated from the 33% of PCC voxels most responsive to meditation (active condition) vs. rest (control condition), enabling dynamic PCC personalisation to accommodate personalised changes in ROI engagement over time and with learning. Specifically, at each time point, PSC was calculated using voxels within the predefined bilateral ventral PCC that showed the strongest response for the meditation < rest contrast. Rest was modelled using a real-time detrended, denoised incremental GLM baseline which reflected the average rest condition up to the given time point. This dynamic approach enabled the feedback calculations to account for individual fMRI signal changes within the target ROI over time and with learning, relative to a stable, detrended baseline representing the cumulative control condition. Real-time physiological control was implemented in MATLAB using cumulative GLM, with PCC PSC, reflecting peak PCC deactivation at each time point, as the response variable and confound ROI PSC, reflecting peak response of the confound ROI to meditation vs. rest contrast, as the predictor. The residualised peak PCC PSC was averaged within each meditation condition (26 s) to estimate the NF score for feedback displayed after the meditation condition (complete details in Online Resource ESM_1.pdf, SM2.7.3). The scores were visualised on a thermometer bar with 20 levels, where higher levels indicated more negative PCC PSC (i.e. deactivation relative to rest) linked to greater meditative focus. Group-level differences in mean real-time residualised PCC PSC signals for meditation vs. rest were assessed using a GLM for each fMRI NF session, controlling for age, sex, and average SSS ratings.

For offline fMRI analyses, all MRI data were preprocessed using fMRIPrep (v23.2.1) (complete details in Online Resource ESM_1.pdf, SM2.9). The offline fMRI preprocessing steps included spatial inhomogeneity distortion correction, non-linear coregistration to anatomical and MNI space, head motion correction, and spatial smoothing with a Gaussian kernel of 2-mm full-width half-maximum (FWHM). Nuisance regressors for blood oxygenation level dependent (BOLD) fMRI denoising included 24 head motion parameters, the top five aCompCor parameters for physiological noise, cosine regressors for high-pass filtering, nine RETROICOR respiration correction regressors, and regressors for non-steady state magnetisation effects from initial fMRI volumes. Overall, these nuisance regressors together captured variance due to MRI artefacts, head motion, and physiological noise. After fMRIPrep quality checks, fMRI data from six participants were discarded due to reduced BOLD signal quality, leaving data from 34 participants (17 experimental, 17 control) for MRI-related analysis.

Activation in the NF target ROI (PCC) during meditation relative to rest was estimated offline through GLM in FSL FEAT (https://web.mit.edu/fsl_v5.0.10/fsl/doc/wiki/FEAT.html; FSL v6.0.6.4). Subject-level GLMs were used to model voxel-wise BOLD responses to meditation, rest, cue, and feedback conditions during each NF run, while controlling for variance associated with the nuisance regressors. Second-level GLM was used to quantify the mean PCC BOLD responses across NF runs for the meditation vs. rest contrast, while controlling for mean framewise displacement (mFD) quantifying average head motion. Group-level differences in mean PCC activation during meditation vs. rest, with age, sex, and average SSS ratings as covariates, were assessed using a third-level GLM for each fMRI session.

In line with the recent neurocognitive network model of focused attention meditation (Ganesan et al., 2022), changes in context-dependent functional negative coupling (i.e. negative correlation between regions during a state) were examined between the NF target ROI (PCC, a core node of the DMN) and all voxels within the SN and CEN during NF-guided meditation vs. rest, using generalised psychophysiological interactions (gPPI) analysis (McLaren et al., 2012) in FSL FEAT and permutation analysis of linear models (PALM) (Winkler et al., 2014). CEN and SN were outlined using the standard seven-network Yeo parcellation (Yeo et al., 2011). The first-level model of the gPPI analysis included the main condition predictors, their respective PPI predictors (PCC BOLD time course × main condition), and the mean PCC BOLD time course, along with the aforementioned nuisance regressors. Second-level modelling was used to calculate the average voxel-wise beta (first-level model fit) values across NF runs within each NF session for the meditation vs. rest contrast, with run-wise mFD values included as covariates. At the third level, group differences per NF session were identified using non-parametric permutation testing (10,000 permutations, uncorrected *p* < 0.005 cluster-forming, family-wise error (FWE) adjusted *p* < 0.05 across clusters). This enabled the identification of brain clusters within the SN and CEN that exhibited significant group differences in their extent of negative functional coupling with the NF target during NF-guided meditation vs. rest. Further details on the analysis pipelines are provided in Online Resource ESM_1.pdf, SM2.11.

Negative functional coupling between PCC and significant NF gPPI cluster/(s) from fMRI data acquired during the pre-NF baseline and post-NF transfer meditation tasks (involving no NF) was computed using Pearson’s correlation functional connectivity (FC), and group differences in FC change scores (transfer FC minus baseline FC) were analysed with GLM (ANCOVA), controlling for mean SSS, mFD, age, and sex.

Offline brain-behaviour associations were examined through Pearson’s partial correlations between each significant behavioural outcome (DASS-21, SMS, and/or BCT measures) and each significant NF target change (PCC activation and/or functional coupling), controlling for age and sex. Variance from mean SSS and mFD was regressed out from NF target changes prior to the correlation analyses. For significant correlations, post hoc group-wise correlations were performed to explore which group drove the observed sample-level correlations. FDR correction (0.05) was applied to control for multiple comparisons across the six PCC-based analyses not involving voxel-wise testing (Online Resource ESM_1.pdf, Table S2).

## Results

### Sample Characteristics

The final sample comprised 40 eligible adults with beginner-level meditation experience and no psychiatric or neurological diagnoses who completed the study (Online Resource ESM_1.pdf, Figure S1). Table 1 depicts the sample characteristics. Participants were assigned to either the experimental group (*n* = 20), which meditated with veridical PCC NF, or a control group (*n* = 20), which meditated with yoked-sham NF based on PCC activity from an experimental participant with similar meditation experience. The participants were blinded to the control group’s existence.

**Table 1:** Key sample and baseline characteristics by group. No significant differences were observed between groups

**Measure**	**Experimental group** **(*n*** **= 20; 13 females)**	**Control group** **(*n*** **= 20; 14 females)**
Ethno-geographic background	Oceania (14), Oceania – North East Asia (1), Americas (2), South Central Asia (1), North West Europe (1), North East Asia (1)	Oceania (8), North East Asia (5), Americas (1), Americas – South East Europe (1), South Central Asia (1), North West Europe (1), South East – North West Europe (1), South East Asia (2)
	**Mean** $\boldsymbol\pm$** standard deviation (median) [minimum – maximum]**	**Mean ** $\boldsymbol\pm$** standard deviation (median) [minimum – maximum]**
Age (years)	29.00$\pm$7.70 (26.0) [19.0–45.0]	29.50$\pm$7.90 (28.0) [19.0–49.0]
Sleep quality over past month: PSQI total scores	5.70$\pm$2.40 (6.0) [1.0–11.0]	5.50$\pm$1.50 (5.0) [4.0–8.0]
Self-reported lifetime meditation experience (hours)	20.80$\pm$45.90 (1.0) [0.0–180.0]	20.00$\pm$44.30 (0.8) [0.0–145.0]
Dispositional mindfulness scores: FFMQ total (***ω*** = 0.63) FFMQ observing (***ω*** = 0.58) FFMQ describing (***ω*** = 0.80) FFMQ acting with awareness (***ω*** = 0.84) FFMQ non–judging (***ω*** = 0.90) FFMQ non–reactivity (***ω*** = 0.80)	3.00$\pm$0.40 (3.0) [2.5–4.4] 3.20$\pm$0.60 (3.1) [2.3–4.4] 3.40$\pm$0.60 (3.5) [2.3–4.3] 2.80$\pm$0.70 (2.7) [1.8–4.6] 3.00$\pm$0.80 (3.1) [1.6–4.5] 2.80$\pm$0.70 (2.6) [2.0–4.6]	3.20$\pm$0.40 (3.3) [2.5–3.7] 3.40$\pm$0.60 (3.5) [2.6–4.5] 3.30$\pm$0.60 (3.5) [2.1–4.3] 3.10$\pm$0.70 (3.2) [2.1–4.3] 3.20$\pm$0.80 (3.2) [1.4–4.4] 2.90$\pm$0.60 (2.9) [1.6–3.6
Dispositional mind-wandering scores: MWQ (***ω*** = 0.85)	4.00$\pm$0.90 (4.0) [2.2–5.4]	3.80$\pm$0.80 (3.9) [2.6–5.0]
Dispositional anxiety scores: STAI-T (***ω*** = 0.65)	2.20$\pm$0.60 (2.3) [1.0–3.1]	2.20$\pm$0.50 (2.1) [1.6–3.4]

Ethno-geographic background classified using typology recommended by the Australian Bureau of Statistics (https://www.abs.gov.au/ausstats/abs@.nsf/Previousproducts/1249.0Main%20Features42011?opendocument&tabname=Summary&prodno=1249.0&issue=2011&num=&view =) *PSQI*, Pittsburgh Sleep Quality Index; *FFMQ*, Five Facet Mindfulness Questionnaire; *MWQ*, Mind-Wandering Questionnaire; *STAI-T*, State and Trait Anxiety Inventory–Trait Module

### Verification of Blinding and Control

Mean sham PCC signals provided to the control group were not significantly correlated (*p* > 0.05) with their actual mean PCC signals in either NF session, after online (NF session 1: *r*(33) = − 0.34, *p* = 0.19; NF session 2: *r*(33) = 0.21, *p* = 0.41) or offline (NF session 1: *r*(33) = 0.08, *p* = 0.76; NF session 2: *r*(33) = − 0.08, *p* = 0.75) fMRI denoising.

*None* of the post-NF self-report ratings, i.e. (i) correspondence between NF scores and the experience of focused attention meditation, (ii) perceived utility of NF for learning meditation, or (iii) in-scanner meditation performance, was significantly different between groups in either NF session (median rating $\ge 3/5$ per group; uncorrected *p* > 0.05; see Online Resource ESM_1.pdf, Figure S3A–S3C), supporting the effectiveness of participant blinding. Notably, this was in addition to neither group being aware of the presence of a control group or sham NF in the study. Participants from both groups reported engaging in focused attention meditation anchored in breathing sensations during meditation conditions, and unconstrained thought during rest conditions (Online Resource ESM_1.pdf, Figure S7).

Post hoc validation testing confirmed the reliability of the online NF signals: PCC deactivation estimated online during NF (adjusted for confound ROI signals) was significantly correlated with PCC deactivation calculated offline (following standard comprehensive offline denoising without using the confound ROI) (*r* = 0.38; *p* < 0.05; see Online Resource ESM_1.pdf, Figure S3D). Furthermore, the signal from the confound region did not significantly differ between groups in either session during meditation vs. rest (NF session 1: *p* = 0.56; NF session 2: *p* = 0.21; see Online Resource ESM_1.pdf, Figure S4B).

Finally, *none* of the baseline measures (i.e. FFMQ and subscales, total DASS-21, STAI-T, PSQI, age, sex, meditation experience, BCT scores, ethno-geographic background) was significantly different between groups (uncorrected *p* > 0.05), suggesting that the groups were equivalent at study commencement.

### Mindful Awareness During 1-Week Real-World Meditation Practice

**Fig. 3:**
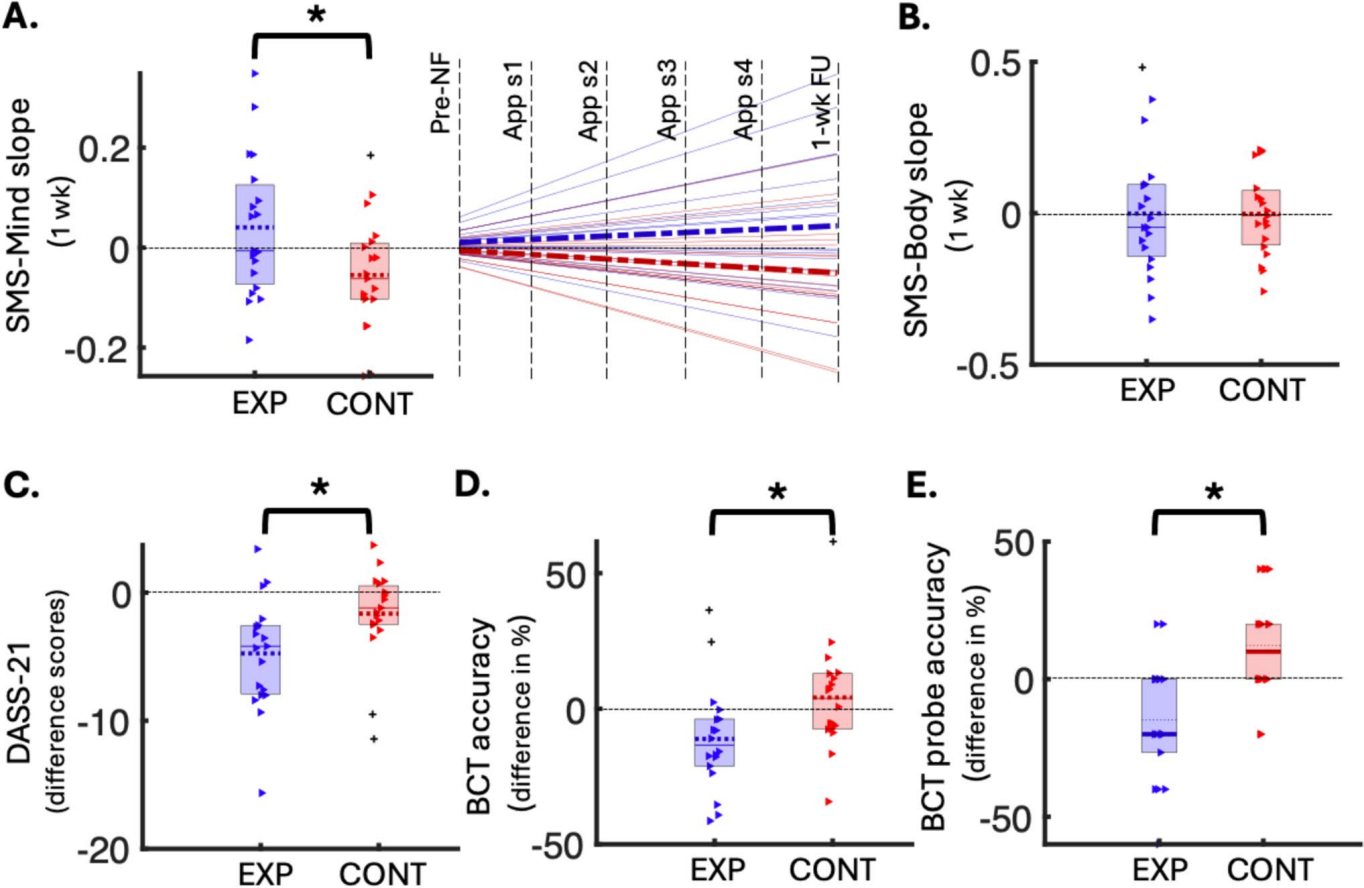
Behavioural outcomes of the NF-guided meditation training. **A **Left: Box plot of group differences in slopes of change (y-axis) in SMS-Mind scores over 1-week meditation practice (5-min sessions) after controlling for age, sex, arousal, and pre-meditation mindful awareness, significant after FDR correction (FDR-*p* = 0.041, uncorrected *p* = 0.019, Cohen’s *d* = 0.38, *n*(exp) = 19, *n*(cont) = 19). Right: Same data represented using a line graph, with the pre-NF data normalised to 0, group mean slopes indicated as bold dashed lines and individual slopes shown as faded solid lines (experimental in blue, control in red). **B** Box plot of group differences in slopes of change (y-axis) in SMS-Body scores over 1-week meditation practice (5-min sessions), not significant (*n*(exp) = 19, *n*(cont) = 19). **C** Box plot showing group differences in DASS-total difference scores (y-axis; follow-up minus baseline), significant after FDR correction (FDR-*p* = 0.041, uncorrected *p* = 0.025, Cohen’s *d* = 0.40, *n*(exp) = 19, *n*(cont) = 19). **D** Box plot of group differences in BCT accuracy changes (y-axis; follow-up minus baseline), significant after FDR correction (FDR-*p* = 0.041, uncorrected *p* = 0.033, Cohen’s *d* = 0.39, *n*(exp) = 18, *n*(cont) = 18). **E** Box plot of group differences in BCT probe accuracy changes (y-axis; follow-up minus baseline), significant after FDR correction (FDR-*p* = 0.005, uncorrected *p* = 0.001, Cohen’s *d* = 0.56, *n*(exp) = 18, *n*(cont) = 18). In all box plots, coloured triangles represent individual data points (red for control, blue for experimental), with outliers shown as pluses. Means are dotted lines and medians are solid lines. EXP, experimental group; CONT, control group; DASS, Depression, Anxiety & Stress Scale; SMS, State Mindfulness Scale; App sx, app meditation session x; wk, week; BCT, breath counting task; NF, neurofeedback; FU, follow-up; *FDR-significant *p* < 0.05

As the week progressed, the control group showed a significantly greater decrease in mindful awareness of thinking during meditation practice compared to the experimental group (Cohen’s *d* = 0.41; FDR-adjusted *p* = 0.04; Fig. 3A), despite equal practice durations. Post hoc within-group one-sample *t*-tests indicated that the SMS-Mind slope in the experimental group was positive but non-significant (*t*(18) = 1.35, *p* = 0.19), and the control group showed a negative slope that trended towards significance (*t*(18) = − 2.15, *p* = 0.05). Trajectories of change in SMS-Mind and SMS-Body scores, with individual data points, are shown in Online Resource ESM_1.pdf, Figure S6, while group-wise SMS-Mind changes over time are depicted in Online Resource ESM_1.pdf, Figure S10. No significant group difference was found for the SMS-Body subscale (Fig. 3B). Mindful awareness during the baseline 5-min meditation session did not significantly differ between groups (*p* > 0.05).

Sensitivity analyses excluding the pre-meditation SMS covariate, but retaining arousal (SSS), age, and sex as covariates, revealed similar results. The SMS-Mind slopes in the experimental group were significantly more positive than in the control group (Cohen’s *d* = 0.50; uncorrected *p* < 0.01; Online Resource ESM_1.pdf, Figure S9A), while no significant differences were found for SMS-Body. Furthermore, analyses of pre-meditation mindful awareness trajectories, controlling for age, sex, and arousal, showed no significant between-group differences in slopes for pre-meditation SMS-Mind or SMS-Body (Online Resource ESM_1.pdf, Figure S9B), indicating that changes in pre-meditation awareness over 1 week were similar across groups and had minimal impact on awareness changes observed during meditation sessions over the 1 week.

### Emotional Distress During 1-Week Real-World Meditation Practice

The experimental group (*M* = − 4.80, *SD* = 5.00 difference score), compared to the control group (*M* = − 1.60, *SD* = 3.6 difference score), exhibited a significantly greater reduction in emotional distress— indicated by a more negative difference score— from baseline to follow-up (Cohen’s *d* = 0.40, FDR-adjusted *p* = 0.04; Fig. 3C). Post hoc within-group one-sample *t*-tests showed that the experimental group’s difference scores were significantly below zero (*t*(18) = − 4.77, *p* < 0.001), while the control group’s scores showed a non-significant trend in the same direction (*t*(18) = − 1.98, *p* = 0.06).

### Breath Counting After 1-Week Real-World Meditation Practice

Contrary to expectations, the experimental group showed a significant decline (i.e. more negative difference scores) in both BCT task performance indices from baseline to follow up, compared to the control group (task-accuracy FDR-adjusted *p* = 0.04; probe-accuracy FDR-adjusted *p* < 0.01; Fig. 3D and E).

The task and probe accuracies were not significantly different between groups at baseline (*p* > 0.05). The breath counting was physiologically valid, as the mean breath rate measured by the respiration belt showed a strong correlation with the mean counting rate (*r* = 0.98, *p* < 0.0001). Post hoc exploratory analyses revealed a significant correlation between increase in mindful awareness during the week-long meditation practice (SMS-Mind slopes) and decrease in BCT task accuracy from baseline to the end of the week-long practice (*r*(33) = − 0.35, *p* = 0.04; Online Resource ESM_1.pdf, Figure S5). Furthermore, at baseline, the BCT task accuracy was not significantly correlated (*p* > 0.05) with dispositional mindfulness (FFMQ and subscales).

### Deactivation of NF Brain Target During NF-Guided Meditation

**Fig. 4:**
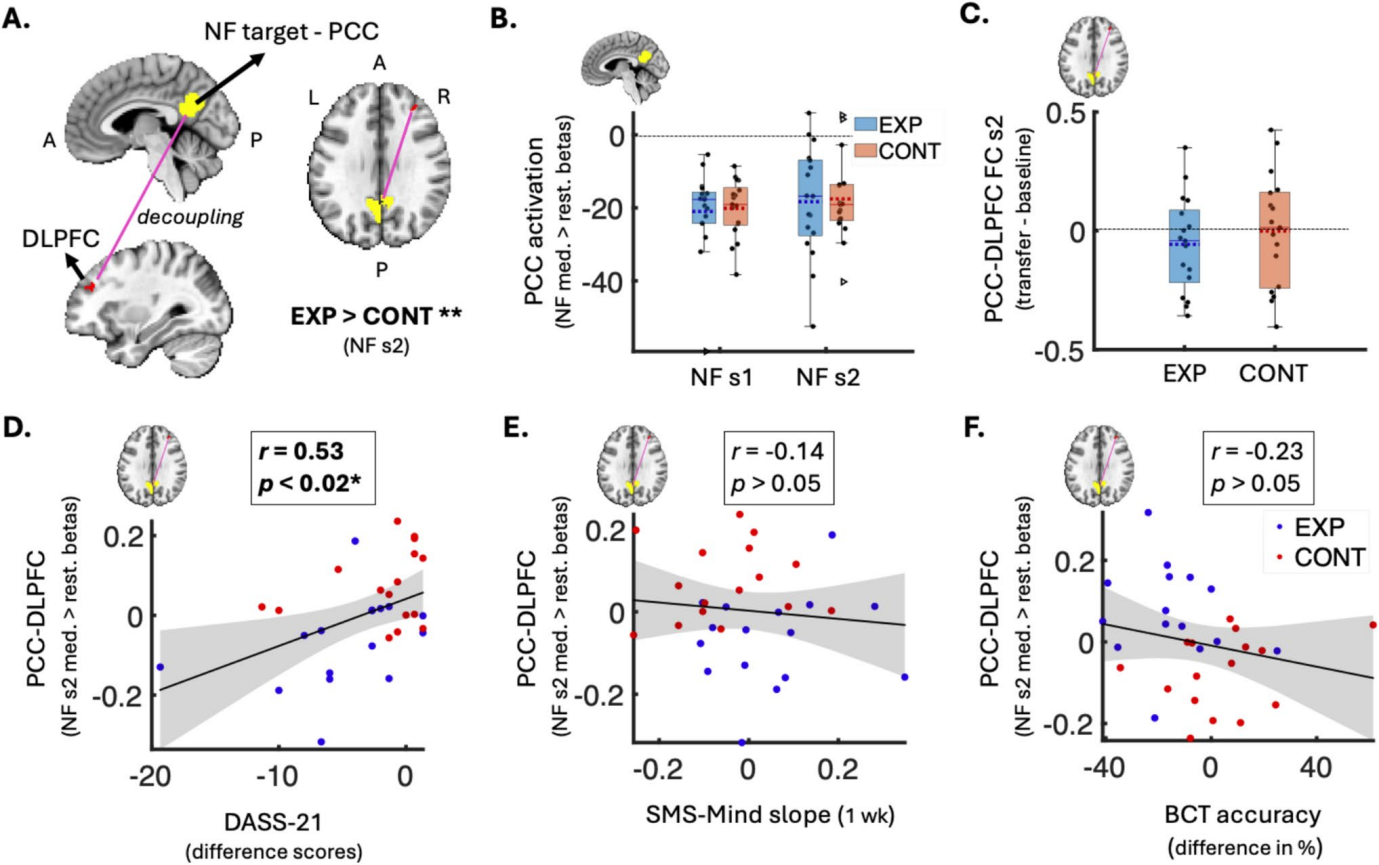
Neural changes and brain-behaviour associations related to the NF-guided meditation training. **A**Brain volume slices showing the negative coupling (pink line) between the NF target (PCC in yellow) and right DLPFC (red; gPPI cluster of 26 voxels) that were significantly stronger in the experimental group vs. control group during NF-guided meditation vs. rest (session 2 FWE-*p* < 0.05, Cohen’s *d* = 0.59, *n*(exp) = 17, *n*(cont) = 17). **B** Box plot of group difference (not significant) in mean PCC activation betas during NF-guided meditation vs. rest in each NF session (*n*(exp) = 17, *n*(cont) = 17). Y-axis represents mean activation values (betas). **C** Box plot of group difference (not significant) in the change in PCC-DLPFC negative coupling (transfer minus baseline) during non-NF meditation in session 2 (*n*(exp) = 17, *n*(cont) = 17). Y-axis represents the difference in FC correlation values. Box plots **B** and **C** show individual data points as black dots, outliers as triangles, data range as whiskers, means as dotted lines, and medians as solid lines. **D** Scatter plot showing a significant positive correlation between increase in PCC-DLPFC negative coupling during NF-guided meditation (y-axis; session 2 gPPI betas) and decrease in emotional distress from baseline to follow-up (x-axis; DASS-Total difference scores) (FDR-*p* = 0.018; uncorrected *p* = 0.003; *r* = 0.53; *n* = 32). **E** Scatter plot showing a non-significant negative correlation between PCC-DLPFC negative coupling (y-axis; session 2 gPPI betas) and SMS-Mind slopes from the 1-week meditation practice (x-axis; SMS-Mind slopes) (*n* = 32). **F** Scatter plot showing a non-significant positive correlation between PCC-DLPFC negative coupling (y-axis; session 2 gPPI betas) and change in BCT accuracy from baseline to follow-up (x-axis; difference in BCT accuracy %) (*n* = 31). In the scatter plots (**D**–**F**), blue dots show experimental group data, red dots show control group data, and grey-shaded areas represent 95% confidence intervals around the linear fits (solid black lines). PCC, posterior cingulate cortex; NF, neurofeedback; s1, session 1; s2, session 2; EXP, experimental group; CONT, control group; DLPFC, dorsolateral prefrontal cortex; gPPI, generalised psychophysiological; med., meditation; rest., restful thinking; FC, functional connectivity; DASS, Depression, Anxiety & Stress Scale; SMS, State Mindfulness Scale; A, anterior; BCT, breath counting task; wk, week; P, posterior; L, left; R, right; I, inferior; S, superior; *FDR-significant *p* < 0.05; **FWE-adjusted *p* < 0.05

No significant differences in offline-denoised PCC deactivation were observed between the groups in either session (*p* > 0.05; Fig. 4B). Similarly, no significant between-group differences were found in the online-estimated, online-denoised PCC PSC values in either session (*p* > 0.05; Online Resource ESM_1.pdf, Figure S7).

### Functional Coupling of NF Brain Target During NF-Guided Meditation

In the first NF session, there were no significant between-group differences (FWE-*p* > 0.05) in negative functional coupling between the PCC target and voxels in SN and CEN.

In the second NF session, we found a significant gPPI cluster (26 voxels) of medium effect size (Cohen’s *d* = 0.59) in the right dorsolateral prefrontal cortex (DLPFC), a core CEN node (FWE-adjusted one-tailed *p* = 0.032; Fig. 4A; Online Resource ESM_1.pdf, Table S2). In other words, relative to rest, the negative coupling between the NF brain target (PCC) and right DLPFC was significantly stronger during veridical NF-guided meditation compared to sham NF-guided meditation in the final training session (day 3 in Fig. 2A). Notably, the between-group difference was driven by significant negative coupling (relative to 0) within the experimental group (*M* = − 0.07, *SD* = 0.11 gPPI beta meditation > rest; one-sample *t*-test: *t*(16) = − 2.54; *p* = 0.02, Cohen’s *d* = 0.63) as well as significant positive coupling (relative to 0) within the control group (*M* = 0.08, *SD* = 0.11 gPPI beta meditation > rest; one-sample *t*-test: *t*(16) = 3.11; *p* < 0.01, Cohen’s *d* = 0.78).

### Change in Functional Coupling of NF Brain Target from Baseline to Transfer Meditation

The change in functional coupling (Pearson’s correlation FC) between the significant gPPI cluster and PCC— from pre-NF baseline meditation to post-NF transfer meditation in the final session containing the NF effects— did not significantly differ between groups (*p* > 0.05; Fig. 4C).

### Relationship Between Behavioural Outcomes and Changes in NF Brain Target

We found that the decrease in emotional distress from the week before baseline to the week of post-NF real-world meditation practice was significantly correlated with the increase in PCC-DLPFC negative coupling during NF-guided meditation (*r*(28) = 0.53, FDR-adjusted *p* = 0.02; Fig. 4D). Post hoc analysis revealed that this association was significant only in the veridical-NF group (*r*(12) = 0.71, *p* < 0.01), but not in the sham-NF group (*r*(12) = 0.06, *p* = 0.83).

The PCC-DLPFC negative coupling was not significantly correlated with the other behavioural outcomes, i.e. change in mindful awareness (*r*(28) = − 0.14; *p* = 0.22; Fig. 4E) or change in breath counting accuracy (*r*(27) = 0.23; *p* = 0.46; Fig. 4F).

Detailed statistics of all main outcomes are provided in Online Resource ESM_1.pdf, Table S2.

## Discussion

We sought to optimise early meditation practice for well-being by implementing a proof-of-concept 7 T fMRI NF-guided meditation training paradigm. This training involved novices learning to meditate from precise and personalised feedback on PCC activity — a key brain region in the DMN implicated in self-referential thought (Leech & Sharp, 2014). The paradigm aimed to assist novice meditators in learning focused attention meditation by training them to deactivate their PCC during meditation relative to rest and to understand how PCC activity relates to meditative states.

Our findings revealed that the experimental group, following two consecutive days of contingent NF-guided meditation training, experienced significantly higher mindful awareness of thinking processes during a week of real-world meditation compared to the control group that received sham NF-guided meditation training (reduced mindful awareness). Additionally, the experimental group experienced a significantly greater reduction in emotional distress over this period of post-NF real-world practice. The magnitude of PCC deactivation during NF-guided meditation vs. rest did not significantly differ between groups on either training day. However, the experimental group exhibited significantly greater negative coupling between the deactivated PCC and the dorsolateral prefrontal cortex (DLPFC), a region crucial for executive functioning and cognitive control (Koechlin et al., 2003), on the final training day. Notably, this increase in PCC-DLPFC negative coupling during NF-guided meditation was significantly associated with the decrease in emotional distress over the week of post-NF real-world meditation practice in the experimental group. No such association was observed in the control group. Sham NF signals provided to the control group were uncorrelated with their actual PCC signals. However, both groups reported similar perceptions of NF authenticity, task performance, and usefulness of NF for learning meditation, suggesting effective blinding and comparable motivation, engagement, expectations, and perception of learning support across groups. This is despite all participants remaining completely unaware of even the existence of a control group or the possibility of receiving sham NF.

The function of the DLPFC in the CEN involves cognitive control, which includes managing attentional resources to facilitate goal-directed behaviour (Koechlin et al., 2003) and amplifying attention to target stimuli while filtering out distractions (Egner & Hirsch, 2005). Negative coupling between CEN regions (e.g. DLPFC) and DMN regions (e.g. PCC) is thought to release attentional resources from sustained thinking, thereby facilitating enhanced bottom-up perception of present-moment sensory and bodily stimuli (Christoff et al., 2009, 2016; Fox et al., 2005; Schooler et al., 2011; Spreng et al., 2010). According to the neurocognitive network model of focused attention meditation (Ganesan et al., 2022), volitionally shifting attention away from default self-referential thought towards target stimuli, such as breathing sensations, is linked to DMN suppression and relative increases in CEN and SN activity. The directionality of activity proposed by this model closely aligns with dynamic FC findings (Mooneyham et al., 2017). Mooneyham et al. (2017) found that more mindful individuals exhibited a greater tendency to occupy a network state with negative coupling between the DMN and both the SN and CEN, while spending less time in a state with positive coupling among these three networks. Furthermore, preliminary evidence suggests that attenuating the DMN relative to CEN through fMRI meditation-NF can positively impact patients with schizophrenia (Bauer et al., 2020) and affective disorders (Zhang et al., 2023). Consistent with these functions of DMN-CEN interaction, our NF-guided meditation training (which targeted PCC (DMN) deactivation in healthy novices) significantly enhanced PCC (DMN)—DLPFC (CEN) negative coupling during focused attention meditation vs. rest on the final training day. Therefore, while the comparable magnitudes of PCC (NF target) deactivation across groups may reflect similar levels of disengagement from self-referential thought during NF-guided meditation, negative coupling of the PCC (NF target) from DLPFC was observed only in the experimental group.

While between-group differences in NF target region activity are typically expected in NF studies, the absence of such differences here does not diminish the value of examining how the target region’s activity was modulated in relation to another key region. Prioritising NF target activation changes alone may miss critical target-related coupling changes. This is especially the case for fMRI NF studies involving meditation—a structured task with well-defined techniques that can be explored through both connectivity and activation measures. Thus, although the NF-guided meditation training was not successful in directly modulating the activation of the targeted brain region relative to the control group, meaningful between-group differences in the target region’s functional coupling were indeed observed. Our functional coupling analysis, seeded at the NF target region (PCC), demonstrated significant group differences in PCC-DLPFC coupling, consistent with the neurocognitive network model of focused attention meditation. Specifically, the PCC-DLPFC negative coupling in the experimental group suggests that this group, compared to the sham NF control group, may have engaged more *intentionally* in attentional control to regulate disengagement from—and consequently enhance awareness of—self-referential thinking (Zhou et al., 2023) beyond their resting baseline. In contrast, positive coupling between the deactivated PCC and DLPFC was observed during sham NF-guided meditation. Such positive coupling suggests that the control group may have entered mind-blanking (characterised by *passive* disengagement from thought and reduced meta-awareness) rather than meditative states, as mind-blanking states can emerge due to fluctuating arousal levels within the MRI scanner (Andrillon et al., 2025). Additionally, the sham NF could have reinforced subjective strategies that are less effective for active disengagement from self-referential thought during meditation, consistent with the lack of clear downstream benefits during subsequent self-guided practice in this group. However, without a third no-NF meditation-only control group, it remains uncertain whether these effects are specific to sham NF or reflect natural patterns of initial learning among meditation novices within an MRI environment (without neurofeedback).

The observed PCC-DLPFC negative coupling during NF-guided meditation vs. rest contrasts with reported effects of meditation on resting-state FC. Resting-state FC effects of meditation typically include PCC-DLPFC positive coupling (Creswell et al., 2016; King et al., 2016; Kral et al., 2019; Sezer et al., 2022) mediated by a positive integration with the awareness-related SN (Bremer et al., 2022; Rahrig et al., 2022). This suggests that training for greater top-down volitional control over self-referential thought during meditation (reflected in negative coupling between *deactivated* PCC and DLPFC) may enhance subsequent resting-state meta-awareness of self-referential thinking (through positive integration of *activated* PCC, DLPFC, and SN).

Sustained meditation practice with effective top-down (volitional) attentional control has been shown to progressively enhance mindful awareness, facilitating a transition to the more effortless, present-centred, and non-reactive attention observed in experienced meditation practitioners (Lutz et al., 2015; Zhou et al., 2023). Early indicators of this process were evident in our study, as the experimental group maintained (and slightly increased) mindful awareness while the control group showed a decline. Specifically, proportional to real-world meditation practice time, the experimental group— which demonstrated stronger NF-driven PCC-DLPFC negative coupling linked to enhanced volitional top-down control— showed sustained (and slightly growing) mindful awareness of thought processes during meditation. In contrast, the control group showed a decline. Thus, continued self-guided meditation practice, augmented by insights and attentional enhancements gained from high-precision NF training, may expedite the development of mindful awareness in practice compared to meditating without the benefits of NF-guided training for the same duration (Goldberg et al., 2014). The between-group differences in mindful awareness change were partly driven by a decline in the sham NF control group. This decline over a 1-week post-NF practice period may reflect early fluctuations in the natural non-linear trajectory of meditation development. The possibility of such non-linear trajectories is consistent with theoretical frameworks (Galante et al., 2023) and empirical evidence from repeated-measures meditation experience sampling (Shoham et al., 2017), such that initial reductions May reverse with extended practice. The veridical NF training May have offset this initial 1-week decline in mindful awareness, instead resulting in sustenance and improvement in mindful awareness with post-NF practice. This suggests that NF training may accelerate progress in meditation practice by preventing early setbacks in awareness and enhancing participants’ ability to quickly overcome moments of low awareness. Further support for this interpretation will require study designs with a higher number of longitudinal measurements for reliable detection of non-linear trajectories. Additionally, it is also possible that sham NF subtly reinforced less effective meditation strategies, hindering improvement in mindful awareness during subsequent practice. Future studies using similar study designs with a no-NF meditation-only control group and several longitudinal measurements are needed to clarify these effects.

In line with our hypothesis, compared to the control group, the experimental group that meditated with greater mindful awareness of thought during the week of real-world meditation practice also experienced greater reduction in negative emotional states during this period. Notably, this reduction in negative emotional states was correlated with the increased NF PCC-DLPFC negative coupling. Within-group analyses further revealed that the reduction in emotional distress was significant only in the experimental group. In the sham NF group, the reduction only trended towards significance. Prior evidence found that a week of meditation practice involving 5-min sessions can mitigate emotional distress in healthy novices (Strohmaier & Goldberg, 2024; Strohmaier et al., 2021). The current study suggests that greater mindful awareness during such 5-min sessions, facilitated by NF-driven improvements in volitional attentional control, can further enhance the impact of week-long meditation practice on emotional well-being. The average reduction in emotional distress in Strohmaier et al. (2021) after a week of 40 min of cumulative practice without any NF training was comparable to the average reduction observed in the sham NF group after a week of 25 min of cumulative practice in the current study. This suggests that even if sham NF in our study subtly reinforced less effective meditation strategies, it did not actively disrupt subsequent meditation practice by increasing distress. In contrast, the extent of mean distress reduction in the veridical NF group after 1 week of practice aligned with the largest magnitude of mean reductions observed after 2 weeks of practice in Strohmaier et al. (2021). This further supports the notion that veridical NF training may accelerate distress reduction by offsetting the naturally slower initial trajectory of meditation’s impact among novices. Overall, meditating with greater mindful awareness of thinking processes can likely facilitate better management of negative emotional states. Such improved emotion regulation is likely achieved through quicker recognition of persistent, negative thought patterns — a key prognostic factor across stress-related disorders like depression (Ehring & Watkins, 2008) — followed by more volitional disengagement from these thoughts.

Contrary to our hypothesis, the experimental group showed a significant decline in breath counting accuracy from baseline to 1-week post-NF, compared to the control group. However, we found that the decrease in breath counting accuracy was significantly correlated with an increase in mindful awareness of thinking processes during the week-long practice. This finding is consistent with recent evidence indicating a negative relationship between breath counting performance and mindfulness (Treves et al., 2025). Additionally, a mega-analysis also found that BCT may not capture mindful attention in beginners (Ching & Lim, 2023). Therefore, it is possible that BCT does not reliably reflect improvements in focused attention meditation associated with NF training. While the NF training emphasised disengaging from self-referential thought (underpinned by DLPFC-PCC negative coupling) to sustain attention on breathing, BCT requires sustaining attention on the counting process (a form of thinking relative to one’s own breath) as well as the breath. Such requirements can introduce a cognitive demand inconsistent with the purely non-cognitive attentional skill reinforced during NF training. Additionally, the experimental group’s ability to accurately count breaths may have diminished due to an increased decentring awareness of cognitive processes facilitated by their post-NF meditation practice. Further validation of these notions is required.

Although the experimental group showed significantly stronger PCC-DLPFC negative coupling during NF-guided meditation on the final training day, the change in PCC-DLPFC coupling from pre-NF baseline to post-NF transfer meditation on that day did not differ significantly between groups. This could be due to multiple factors. Firstly, an hour-long MRI scanning session with fMRI-NF training can induce substantial mental fatigue (May et al., 2020). Strong mental fatigue may have hindered transfer effects, particularly among novices who already require substantial attentional effort to meditate. Secondly, the effects of fMRI-NF often emerge gradually rather than immediately post-NF (Rance et al., 2018). Consistently, in the current study, significant outcomes emerged over the course of a week. Postponing the transfer task for a follow-up session could enhance sensitivity to neuronal transfer effects.

Previous fMRI meditation-NF studies with small samples of clinical and healthy novices have reported preliminary evidence of an increase in state mindfulness (Kirlic et al., 2022; Zhang et al., 2023), decrease in auditory hallucinations (Bauer et al., 2020), and improvement in attention (Pamplona et al., 2020) post-NF. However, most of these studies lacked control groups, adjustments for multiple comparisons, real-time physiological denoising, or follow-up assessments, and used continuously updated visual NF (i.e. feedback updated every TR).

In the current paradigm, we included a yoked-sham control group that followed the same strategies and training as the experimental group, except for the differing feedback contingencies. Crucially, neither group was informed of even the existence of a control group. Therefore, control over non-specific factors like motivation, expectancy, reward, and task strategy was likely well managed. The comprehensive control likely facilitated effective isolation of the effects of learning to meditate by *intentionally* regulating attention using guidance from the NF target (Lubianiker et al., 2019; Sorger et al., 2019). As a proxy for online physiological correction during NF, we used a control brain region spanning multiple areas normally influenced by physiological responses (Birn et al., 2006). This likely allowed participants to engage more with mindful attention rather than breath control while learning meditation through NF modulation (Weiss et al., 2020).

Alongside a 1-week behavioural follow-up, we incorporated repeated ecological sampling of real-world meditation. The repeated ecological sampling likely increased sensitivity to the evolving benefits of high-precision NF-guided training in natural settings, thereby enhancing the paradigm’s translational value (Krause et al., 2024). During NF training, participants received intermittent feedback after short meditation intervals to address the drawbacks of continuous NF paradigms. The major drawback of continuous NF is the rise in neurocognitive load during task performance, due to participants having to simultaneously monitor and process the NF signal, haemodynamic delays, and fluctuating visual inputs (Lubianiker et al., 2019; Stoeckel et al., 2014). The intermittent NF in our study may have supported novices by minimising distractions associated with continuous NF during meditation, though the impact of intermittent vs. continuous NF on meditation needs further investigation.

In our study, the intermittent feedback, though averaged from meditation blocks, accounted for the voxel-wise, time-varying dynamics of PCC deactivation within each block. The higher spatiotemporal resolution and signal-to-noise ratio of 7-T fMRI (Pohmann et al., 2016; Torrisi et al., 2018), compared to commonly used 3-T fMRI, likely enhanced the reliability of these neuroanatomically focal signal dynamics and thereby the feedback. The specificity of 7-T fMRI also extended to behavioural effects. The NF training, which involved modulation of the PCC linked to self-referential thought, specifically enhanced mindful awareness of thinking processes but not bodily sensations during subsequent real-world meditation. Finally, we have ensured methodological transparency by adhering to the CRED-nf checklist (Ros et al., 2020).

In NF paradigms, participants may receive explicit instructions on how to regulate their brain activity or be encouraged to implicitly discover effective strategies independently (Paret et al., 2019; Watanabe et al., 2017). The current study employed a predominantly explicit NF approach. Participants were informed about the NF signal’s source, its relevance, and the strategy (focused attention meditation), with some flexibility in technique application. The increase in DLPFC-PCC negative coupling on the final training day suggests improved top-down volitional attentional control over PCC-related thought processes. However, it does not guarantee that participants, particularly novices with limited introspective awareness (Fox et al., 2012), were explicitly aware of all the nuanced subjective strategies driving these attentional effects. Hence, novices may not immediately recognise NF’s utility for learning meditation. Similarly, continued self-guided meditation practice during the subsequent week may have reinforced some of the learnings from NF training, without necessarily enabling full awareness of all the cognitive and experiential processes driving the outcome. Future studies should include detailed longitudinal phenomenological assessments to better illuminate the conscious and subconscious processes involved in NF-guided meditation.

### Limitations and Future Directions

Although a robust control group is included, the study is exploratory with a modest sample size, necessitating cautious interpretation of the findings. The study employed single-blinding (participant-blinding) which may have introduced experimenter biases. Although participant self-reports suggested successful participant blinding, future research should aim for pre-registered, double-blinded RCTs to robustly validate the efficacy of NF-guided meditation training. Longer-term follow-ups with intensive repeated-measures sampling can be valuable. Such follow-ups can help track enduring linear and non-linear changes in meditation quality, dispositional mindfulness, and well-being, as well as assess the reliability of SMS in capturing meaningful day-to-day fluctuations. Although both groups received identical instructions, potential stagnation or worsening due to sham NF training could have influenced the observed group differences. Future studies with a no-NF meditation control group in addition to the sham NF control group should be conducted. Specifically, an additional no-NF group can help explore whether the observed improvements from veridical NF training can be extended beyond those from non-NF self-guided, teacher-guided, or app-based meditation practice. Similarly, future studies with task-design baseline and transfer sessions are suggested. Task-design baseline and transfer sessions can help identify changes in target region activation in the absence of feedback, paralleling activation outcomes from the NF sessions. This approach was not feasible in the current study due to the resting-state-style design of baseline and transfer sessions.

In conclusion, we demonstrated promising evidence that meditation training guided by high-precision NF, compared to meditation training with sham NF, can increase *intentional* control over disengagement from self-referential thinking processes. This, in turn, can lead to sustained and growing mindful awareness of thinking processes during subsequent real-world meditation practice and greater emotional well-being benefits from this practice. Our findings also underscore the importance of ecological sampling in NF paradigms to advance NF’s translational therapeutic value.

## Supplementary Information

Below is the link to the electronic supplementary material.

ESM1 (PDF 1.22 MB)

## Data Availability

Upon request and necessary ethics approvals from the requester, data will be deidentified and shared. Code used for real-time fMRI-NF, and offline data analysis can be accessed via the project’s Github repository (https://github.com/saampras/Melbourne-7T-fMRI-Neurofeedback-guided-meditation/tree/main).

## Declarations

### Use of Artificial Intelligence

Artificial Intelligence (AI) tool ChatGPT was used solely for grammatical editing of some sections. No AI tools were employed to *generate* any of the content presented in this paper.
