# Supplementary material for: Neurofeedback Training Facilitates Awareness and Enhances Emotional Well-being Associated with Real-World Meditation Practice: A 7-T MRI Study
Source: Mindfulness (N Y). 2025 Sep 26;16(10):2787–807. doi: 10.1007/s12671-025-02671-z (PMC12660472; doi:10.1007/s12671-025-02671-z)
Supplement: Supplementary file 1 — (PDF 1.22 MB) [file 12671_2025_2671_MOESM1_ESM.pdf]

Supplementary Material for

Neurofeedback training facilitates awareness and enhances emotional well-being associated with real-world meditation practice: A 7 Tesla MRI study

This file includes

Figure S1: 3
Table S1: 3
SM2 Study Procedure 6
SM2.1 Pre-baseline 6
SM2.2 Baseline with self-guided meditation (Day 1) 6
SM2.3 fMRI neurofeedback (NF) - guided meditation training (Day 2 & Day 3) 7
SM2.4 Self-guided meditation at home (during week after NF) 7
SM2.5 Follow up with self-guided meditation (1 week after NF) 8
SM2.6 MRI data acquisition 8
SM2.7 Real-time fMRI 8
SM2.7.1 Acquisition 8
SM2.7.2 Preprocessing 9
SM2.7.3 Feedback calculations 9
SM2.8 fMRI NF-guided meditation training procedure 10
Figure S2: 13
SM2.9 Offline MRI preprocessing 14
SM2.10 Offline behavioural data analysis 15
Change in mindful awareness during real-world meditation 15
SM2.11 Offline fMRI data analysis 16
SM2.11.1 GLM activation analyses 16
SM2.11.2 gPPI coupling analysis 16
Figure S3: 17
Figure S4: 18
Figure S5: 19
Table S2: 19

Figure S6: 21
Figure S7: 22
Figure S8: 22
Figure S9: 23
Figure S10: 24
References 25

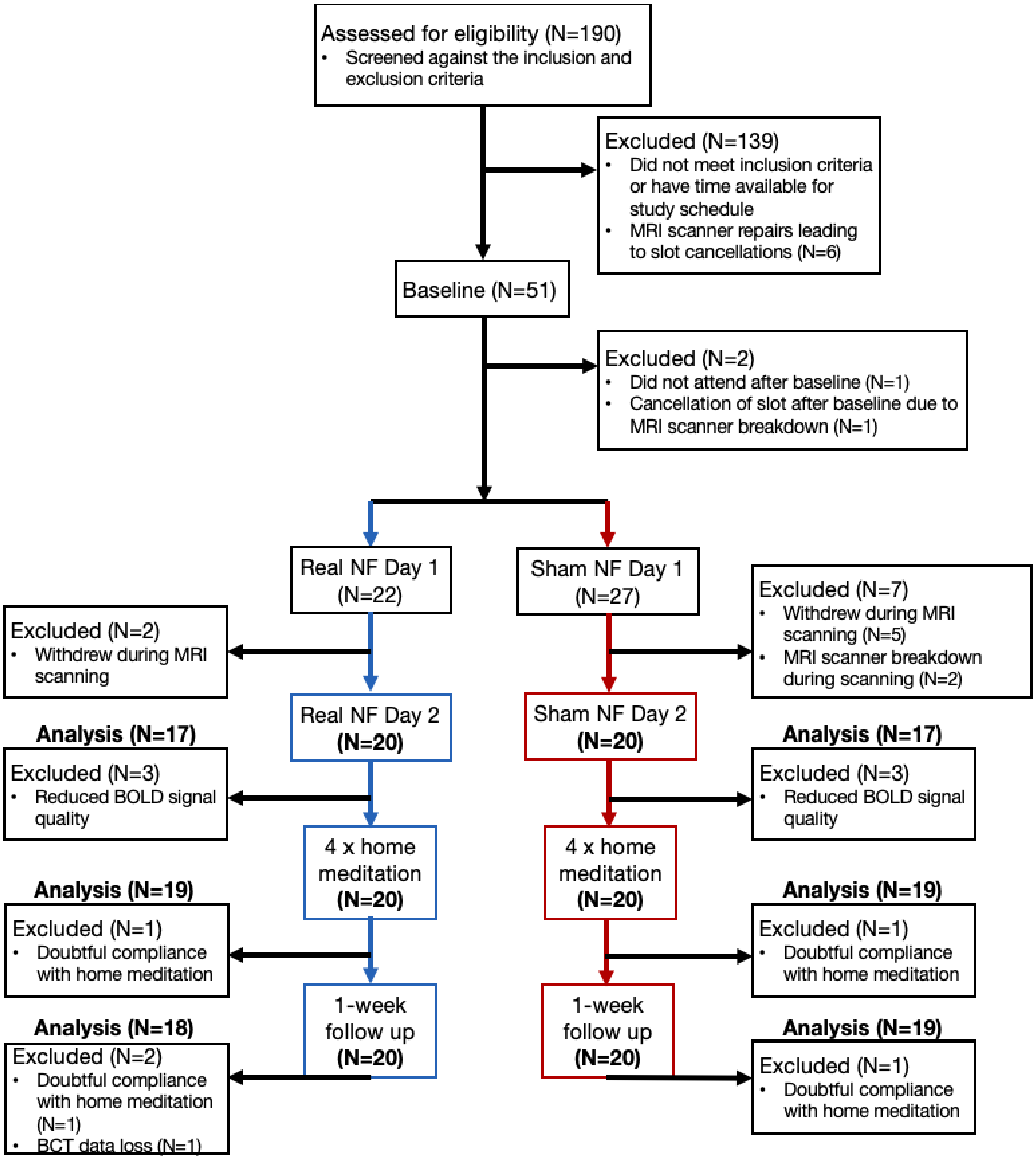

Figure S1:

CONSORT schematic illustrating participant recruitment, dropout, completion, and data inclusion for analysis.

Table S1:

Consensus on the Reporting and Experimental Design of clinical and cognitive-behavioural Neurofeedback studies (CRED-nf) best practices checklist (Ros et al., 2020)

Domain	Item no.	Checklist item	Reported in section in main manuscript
**Pre-experiment**			
	1a	Pre-register experimental protocol and planned analyses	N/A - proof-of-concept study
	1b	Justify sample size	N/A - proof-of-concept study
**Control groups**			
	2a	Employ control group(s) or control condition(s)	METHOD - Sample, Study design
	2b	When leveraging experimental designs where a double-blind is possible, use a double-blind	N/A - proof-of-concept study was single-blind (participants were blinded, and unaware of even the presence of a control group)
	2c	Blind those who rate the outcomes, and when possible, the statisticians involved	N/A
	2d	Examine to what extent participants and experimenters remain blinded	METHOD - Sample
	2e	In clinical efficacy studies, employ a standard-of-care intervention group as a benchmark for improvement	N/A - proof-of-concept study in healthy adults
**Control measures**			
	3a	Collect data on psychosocial factors	RESULTS - Sample characteristics, Table 1
	3b	Report whether participants were provided with a strategy	METHOD - MRI sessions with neurofeedback
	3c	Report the strategies participants used	RESULTS - Verification of blinding and control
	3d	Report methods used for online-data processing and artefact correction	METHOD - Statistical Analyses - Real-time fMRI
	3e	Report condition and group effects for artefacts	RESULTS - Verification of blinding and control
**Feedback specifications**			
	4a	Report how the online-feature extraction was defined	METHOD - Statistical Analyses - Real-time fMRI
	4b	Report and justify the reinforcement schedule	Introduction (Pg 7-8); METHOD - Study design - MRI sessions with neurofeedback; METHOD - Statistical Analyses - Real-time fMRI; DISCUSSION (Pg 42)

	4c	Report the feedback modality and content	METHOD - Study design - MRI sessions with neurofeedback; METHOD - Statistical Analyses - Real-time fMRI
	4d	Collect and report all brain activity variable(s) and/or contrasts used for feedback, as displayed to experimental participants	METHOD - Statistical Analyses - Real-time fMRI, Offline MRI, Offline neuro-behavioral associations; RESULTS - Changes associated with the NF brain target, Relationship between behavioural outcomes and changes in NF brain target
	4e	Report the hardware and software used	METHOD - Study design - MRI sessions with neurofeedback
**Outcome measures**			
Brain	5a	Report neurofeedback regulation success based on the feedback signal	RESULTS - Changes associated with the NF brain target
	5b	Plot within-session and between-session regulation blocks of feedback variable(s), as well as pre-to-post resting baselines or contrasts	Figure 4
	5c	Statistically compare the experimental condition/group to the control condition(s)/group(s) (not only each group to baseline measures)	RESULTS - Changes associated with the NF brain target
Behaviour	6a	Include measures of clinical or behavioural significance, defined a priori, and describe whether they were reached	METHOD - Statistical Analyses - Behavioral data; RESULTS - Behavioural outcomes of NF training
	6b	Run correlational analyses between regulation success and behavioural outcomes	METHOD - Statistical Analyses - Offline neuro-behavioural associations; RESULTS - Relationship between behavioural outcomes and changes in NF brain target
**Data storage**			
	7a	Upload all materials, analysis scripts, code, and raw data used for analyses, as well as final values, to an open access data repository, when feasible	Code is available on Github. Data will be shared upon request and satisfaction of required ethics approvals. Data will also be shared with global consortia such as ENIGMA-Meditation and ENIGMA-Neurofeedback.

Darker shaded boxes represent *Essential* checklist items; lightly shaded boxes represent *Encouraged* checklist items.

SM2 Study Procedure

SM2.1 Pre-baseline

During the week before baseline measurements, participants completed two five-minute audio-guided sessions of focused attention meditation provided by the Epworth Clinic (https://www.epworth.org.au/our-services/mental-health/resources). These sessions familiarised participants with the meditation technique. Participants used the mobile Ecological Momentary Assessment (mEMA) app (https://ilumivu.com/solutions/mobile-health/) on their phones, completing one session per day at home. The mEMA approach captures real-world experiences closer to when and where they occur, reducing response biases and memory distortions.

SM2.2 Baseline with self-guided meditation (Day 1)

On day 1, participants completed various assessments: FFMQ for dispositional mindfulness, MWQ for dispositional mind-wandering, STAI-T for dispositional anxiety, PSQI for 1-month sleep quality, BCT for breath counting skill, and DASS-21 for 1-week emotional distress. They then engaged in a 5-minute session of self-guided, silent, eyes-closed meditation with focused attention on their breath, following instructions adapted from literature (Arch & Craske, 2006).

The instructions were as follows:

"Sit comfortably and upright. Close your eyes and focus on the actual sensations of breath entering and leaving the body. There is no need to think about the breath, no need to change or control it. Just experience the sensations of it as you naturally breathe in and out. Whenever you notice that your awareness is no longer on the breath and you have been distracted by thoughts, emotions or other sensations, gently bring your awareness back to the sensations of breathing. You will hear a bell ring to mark the start and end of your session."

Before and after the meditation, participants completed the SMS and SSS. The pre-meditation SMS measured mindful awareness during the 5 minutes before the session, while the post-meditation SMS measured mindful awareness during the meditation period.

SM2.3 fMRI neurofeedback (NF) - guided meditation training (Day 2 & Day 3)

One day after the baseline, participants began fMRI NF-guided meditation training, which included two sessions over consecutive days (one session per day). NF-guidance for meditation was based on BOLD activity in the posterior cingulate cortex (PCC).

SM2.4 Self-guided meditation at home (during week after NF)

During the week after NF-guided meditation training, participants used the mEMA app at home to complete four 5-minute sessions of self-guided, silent, and eyes-closed focused attention meditation, while applying insights from their NF training.

The instructions were as follows:

"

1. *Please find a comfortable place and posture.*
2. *You will hear a bell ring in the audio to mark the start and end of your session.*
3. *You may use earphones/headphones for this session. If using, please connect it to your phone before proceeding.*
4. *To meditate, you will follow the same basic technique you practised during the fMRI neurofeedback training.*
5. *Sit comfortably and upright.*
6. *Focus on the sensations you feel in your abdomen (belly and chest area) as you breathe normally.*
7. *Try not to take deeper or shallower breaths than usual.*
8. *Whenever you notice your mind being distracted by thoughts, emotions or other sensations, gently acknowledge and bring your focus back to the abdomen.*
9. *You can keep your eyes closed during the session.*
10. *Start meditating when you hear the bell in the audio. You can stop meditating when you hear the next bell after 5 minutes.*

"

Although participants were advised to focus on breathing sensations at the abdomen (similar to the NF sessions), they were also free to instead direct their attention to other areas of the body associated with breathing sensations if those felt more convenient. Before and after each session,

participants completed the SMS and SSS questionnaires to measure mindful awareness before and during meditation. They were required to complete four sessions, with no more than one per day, and could schedule them flexibly. Text reminders were sent to promote adherence.

SM2.5 Follow up with self-guided meditation (1 week after NF)

Approximately one week after the NF training, participants visited the laboratory for the final 5-minute session of self-guided, silent, and eyes-closed meditation (following same instructions as above), with SMS and SSS administered before and after. They were also administered the DASS-21, to examine emotional distress over the past week, and the BCT, to evaluate breath counting skill.

Some secondary measures were also acquired at different time points, including pre- and post-NF SMS, go/no-go task and working memory task, which will be reported separately.

SM2.6 MRI data acquisition

MRI data was acquired on a 7 Tesla MRI scanner (Siemens Magnetom 7T plus) at the Melbourne Brain Centre Imaging Unit (MBCIU) using an 8/32 PTX/RX channel head coil. High-resolution T1-weighted (T1w) anatomical images (3D-MP2RAGE; 0.75mm×0.75mm×0.75mm; TE/TR=2ms/5000ms) were denoised and corrected for Radio Frequency (RF) inhomogeneity. Whole-brain functional images (1.6mm×1.6mm×1.6mm; TE/TR=22ms/800ms; multiband acceleration=6; field-of-view=208mm; matrix size=130×130; 84 slices; slice thickness=1.6mm; flip angle=45^0; P-A phase encoded) were acquired every 800 ms using a multiband gradient-echo echo-planar imaging (EPI) sequence. An MRI compatible two-button response box was used for participants to provide behavioural ratings (SSS) inside the scanner.

SM2.7 Real-time fMRI

SM2.7.1 Acquisition

The Turbo-BrainVoyager software (version 4.2; Brain Innovation, Maastricht, the Netherlands), installed on a dedicated computer, was used for real-time fMRI processing. MATLAB Psychtoolbox (version 3.1), installed on a separate computer, managed the timed visual display of cues, instructions, and feedback presentation during MRI scanning. The Siemens MRI console

computer received the reconstructed MRI DICOMs. All three computers - the one running Turbo-BrainVoyager, the one using Psychtoolbox, and the MRI console computer - were connected to a common network via Transmission Control Protocol (TCP). Reconstructed MRI DICOM images were exported from the MRI console computer via the Siemens IDEA command tool to Turbo-BrainVoyager in real-time. NF scores were calculated and visualised in the computer running MATLAB and Psychtoolbox, using outputs received in real-time from Turbo-BrainVoyager.

SM2.7.2 Preprocessing

Following anatomical scan and prior to fMRI scanning, skull stripping and brain image extraction were performed on the anatomical image in Turbo-BrainVoyager. Real-time fMRI preprocessing in Turbo-BrainVoyager included: linear coregistration of fMRI reference volume (run's first volume) with the anatomical and subsequently the Montreal Neurological Institute (MNI) standard stereotactic space, motion correction with 3D trilinear interpolation (realigning each fMRI volume to reference volume), and spatial smoothing (Gaussian kernel of 3.2 mm full width at half maximum). Additionally, real-time voxel-wise linear detrending and 6-parameter head motion regression were performed through incremental general linear modelling (iGLM) to correct for linear and non-linear confounds affecting the BOLD signal. Real-time physiological control was also implemented through cumulative GLM (cGLM) regression with the BOLD signal from the confound ROI in MATLAB.

SM2.7.3 Feedback calculations

For each meditation condition (32 TRs long), the NF score (ns) was calculated from the mean residual peak PSC values of the PCC (p) across timepoints (TRs) within that condition, excluding the initial 12 TRs (9.6 seconds). Specifically, seven initial TRs were excluded to account for hemodynamic lag, and five initial TRs were excluded to account for transitioning into the meditative condition. The score was then derived from the mean of the remaining 20 TRs, using a standard PSC threshold (thr) of -2%, on an integer scale of 1-20. The neurofeedback score for each meditation condition was estimated in MATLAB as follows:

$$ns = ([\sum_{p=t+e+1}^{t+n} p/(n-12)] \, / \, thr) \times 20 \, ,$$

Where,

e denotes the number of initial timepoints within the meditation condition that were excluded during calculations,

n denotes the total number of timepoints within the meditation condition,

t denotes the total number of timepoints up to the start of the meditation condition.

Negative scores and zeros were adjusted to 1, and positive scores greater than 20 were capped at 20. This ensured an increase in NF scores exclusively reflected negative PSC (i.e., deactivation) in the PCC during meditation compared to resting baseline.

SM2.8 fMRI NF-guided meditation training procedure

Participants completed two identical fMRI NF-guided meditation training sessions over consecutive days. They were instructed to avoid caffeine for two hours before each session.

Prior to the first MRI session, participants were introduced to the meditation and control conditions, as well as the visual NF interface, in a mock scanner. They were thoroughly briefed on the NF paradigm and meditation technique to ensure adequate familiarisation.

The debriefing instructions prior to entering the scanner were as follows:

"

1. *Meditation with eyes open lying inside the MRI scanner can be difficult for some people. But training to focus attention on your abdomen sensations despite this difficulty can potentially strengthen your attention and meditation skills.*
2. *The feedback score represents your average level of focus and awareness of breathing sensations. This means that consistently good focused attention and awareness throughout a meditation period is likely associated with higher scores, compared to short instances of good or very good focused attention and awareness.*
3. *Try to use the MRI training to learn and become more aware of these associations from direct experience of meditation.*
4. *Use the feedback display from each meditation period as a guidance to really understand and evaluate what it feels like to be optimally focused and aware, and not optimally focused and aware. You can then try to apply this insight during meditation without neurofeedback.*
5. *During rest periods, do not meditate. You can think about whatever comes to mind. Some examples: planning your day/week; thinking about your memories, family, friends, etc.; thinking about adjectives that describe your personality; etc.*
6. *During meditation periods, focus and observe the sensations and feeling of breathing, for example at the abdomen (part in contact with the belt). Examples of sensations include tightness, warmth, coolness, tingling, touch, etc.*

7. *During meditation, the <u>quality of focus and awareness is higher when distractions are lower</u>. Distractions include thinking, day-dreaming, stories, imagination, narratives, interpretations, etc.*
8. *Please try to <u>keep your head as still as possible</u> during each scanning session. Head movements can negatively impact MRI signal, and sometimes affect your feedback scores.*
9. *Go in with an open mind and be patient.*

"

Participants were advised to focus on breathing sensations at the abdomen, as it could have facilitated a clear perception of sensations due to the physical contact of the respiration belt. However, they were also encouraged to instead direct their attention to other areas of the body associated with breathing sensations if those felt more convenient.

During the anatomical scan before fMRI measurements, participants had another practice NF run for familiarisation. The first fMRI run involved a baseline meditation task without NF (self-guided meditation) for 2.5 minutes, with instructions to keep their eyes open and attend to breathing sensations in the abdomen area while breathing normally.

The instructions provided during the fMRI sessions inside the scanner were as follows:
<u>Baseline meditation task (no NF):</u>
"Please keep your eyes open throughout the session. You will NOT receive neural feedback for this session. To MEDITATE: Focus on the sensations and feelings in your stomach area while you breath normally as usual. There is NO need to think about the sensations or change it. Just experience the sensations and feelings as they happen. Whenever you notice that your awareness is no longer on the stomach area, gently bring your awareness and focus back to the sensations of your stomach area. When you see a grey cross on the screen, keep your eyes fixed on the cross and start meditation. Breathe normally as usual."

<u>Meditation condition within NF runs</u>
"To MEDITATE: Keep your eyes open. FOCUS on the sensations in your stomach area when the cross appears. Breathe normally as usual. You will see feedback on your performance after each meditation period."

Rest condition within NF runs

"Keep your eyes open. THINK whatever comes to mind freely, when the cross appears. Breathe normally as usual."

Transfer meditation task (no NF)

"Please keep your eyes open throughout the session. You will NOT receive neural feedback for this session. You will meditate by applying what you have learnt during the neurofeedback training so far. The technique of meditation is the same. When you see a grey cross on the screen, keep your eyes fixed on the cross and start meditation. Breathe normally as usual."

After the baseline meditation run, participants completed three identical fMRI NF runs (710 TRs each) with a blocked design: three blocks of rest (51s each) and six blocks of focused attention meditation (26s each) (**Figure 1B** - main text). Visual NF was displayed intermittently after each meditation block, showing the average PCC PSC from the most recent block and a history of previous scores (**Figure S2**). In each NF run, there were three pairs of meditation and NF blocks between each pair of rest blocks. Short 26-second meditation blocks were used based on previous evidence that they are suitable for novice meditators in fMRI tasks (Ganesan et al., 2023).

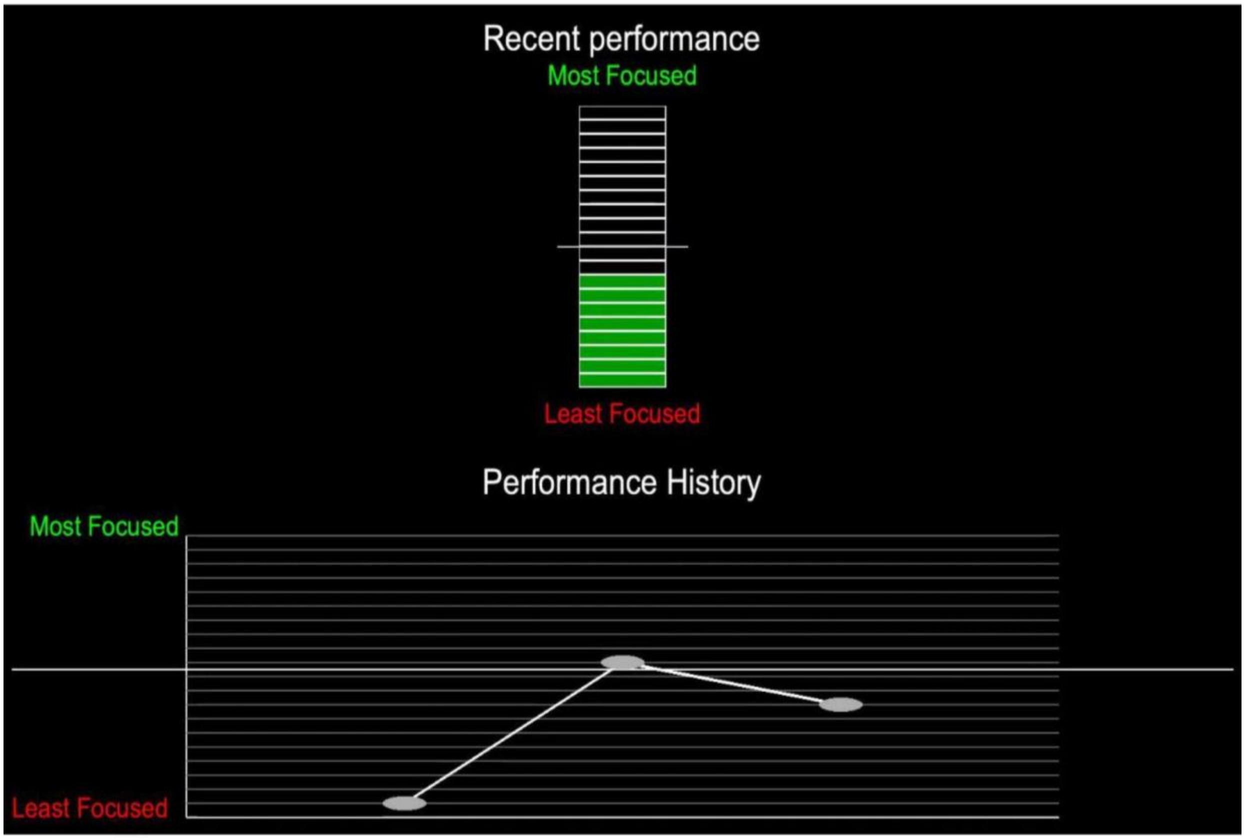

Figure S2:

The visual NF display shown to participants intermittently during the fMRI NF-guided meditation training. Presented for approximately 10 seconds at the end of each meditation block, the display featured a bar under 'recent performance' representing the NF score from the recently concluded meditation block. Participants were instructed to fill up the bar through focused attention meditation. Under 'performance history', a line graph displayed scores from the last two meditation conditions alongside the current one, offering participants context on their performance relative to recent trials.

The final fMRI run, after the NF runs, involved a transfer meditation task identical to the baseline meditation task, without NF, for 2.5 minutes (188 TRs). After each fMRI run, participants used buttons to rate their current level of sleepiness via the SSS. Participants kept their eyes open throughout the fMRI sessions.

Throughout the NF training, participants were instructed to maintain normal breathing during meditation to minimise confounding effects of breathing variations that can negatively affect

meditation fMRI studies (Ganesan et al., 2022, 2023). Such variations in breathing could otherwise encourage and teach participants to employ breath control instead of the intended meditation technique to increase their NF scores.

SM2.9 Offline MRI preprocessing

All MRI data was converted to BIDS format (v1.4.0) using the dcm2niix tool (version 1.0.20230411). Preprocessing was implemented in fMRIPrep 23.2.1.

For the T1w anatomical images, intensity non-uniformity correction and skull stripping were performed using Advanced Normalisation Tools (ANTs) (Avants et al., 2009). FSL FAST was used to segment the brain-extracted T1w images into cerebrospinal fluid (CSF), white matter (WM) and grey matter (GM) (Smith et al., 2004). For each participant, the brain-extracted T1w images from the two MRI sessions were merged into a single unbiased T1w image (equidistant from both the source T1w images) using FreeSurfer's mri_robust_template (Reuter et al., 2012). The merged T1w images were spatially normalised to the standard space (MNI152NLin2009cAsym) using ANTs volume-based non-linear registration.

For each fMRI run per subject, the following preprocessing steps were performed in fMRIPrep (Esteban et al., 2019). An EPI BOLD reference volume was generated. Spatial distortions in the EPI BOLD images caused by B0 magnetic field inhomogeneities were corrected by estimating fieldmaps using FSL Topup (Andersson et al., 2003), utilising opposite phase-encoded (P-A and A-P) spin-echo EPI images acquired during each fMRI session. The estimated fieldmaps were then aligned with the EPI BOLD reference volume using rigid-registration. The distortion-corrected EPI BOLD reference was co-registered to the T1w reference using boundary-based registration with six degrees of freedom in FreeSurfer (Greve & Fischl, 2009). Head-motion parameters relative to the EPI BOLD reference, including transformation matrices and six rotation and translation parameters, were estimated before spatiotemporal filtering using FSL mcflirt (Jenkinson et al., 2002). All resampling was done with a single interpolation step, resulting in preprocessed, spatially normalised fMRI BOLD data. Spatial smoothing was then applied to the preprocessed data using a Gaussian kernel with a 2 mm full-width half-maximum (FWHM) in FSL.

The smoothed preprocessed BOLD data was denoised using nuisance regressors. Specifically, the nuisance regressors included 24 parameters for head motion correction (six head motion parameters, their derivatives and squared derivatives), the top five anatomical component-based

noise correction (aCompCor) parameters for physiological noise correction (Behzadi et al., 2007), cosine regressors for high-pass temporal filtering, nine regressors for respiration correction (including one for respiratory volume change) estimated from respiration belt data using RETROspective Image CORrection (RETROICOR) within the PhysIO toolbox (Kasper et al., 2017), and regressors for the initial fMRI volumes with non-steady state magnetization effects.

SM2.10 Offline behavioural data analysis

Change in mindful awareness during real-world meditation

We quantified the change in trajectory (i.e., slope) of mindful awareness during self-guided meditation using SMS scores collected immediately after each of the six five-minute sessions. The first meditation session was conducted at baseline before NF training.

To account for non-specific fluctuations in transient baseline mindful awareness before meditation and sleepiness during the meditation period, pre-meditation SMS scores and mean SSS ratings were included as covariates. Specifically, to isolate the change in mindful awareness during meditation practice, we regressed out pre-session SMS scores ($Ysmspre_t$), mean SSS ratings ($Ysss_t$), age ($Yage_t$) and sex ($Ysex_t$) from the post-session SMS scores ($Ysmspost_t$) at each time point t, thereby removing variance related to baseline mindful awareness, sleepiness and participant-specific characteristics from each session (eq. 1).

$$Yres_t = Ysmspost_t - (\beta_{1,t} Ysmspre_t + \beta_{2,t} Ysss_t + \beta_{3,t} Yage_t + \beta_{4,t} Ysex_t) \quad --(1)$$

$\beta_{1-4,t}$ denote parameters of the covariate predictors in eq. (1).
Using the residuals ($Yres_{1-6}$) from eq. (1), we performed repeated measures ANCOVAs for each participant across the six timepoints, with time since baseline (T_{1-6}) as the predictor variable (eq. 2).

$$\begin{bmatrix} Yres_1 \\ Yres_2 \\ Yres_3 \\ Yres_4 \\ Yres_5 \\ Yres_6 \end{bmatrix}_p = \beta_0 + \beta_{1,p} * \begin{bmatrix} T_1 \\ T_2 \\ T_3 \\ T_4 \\ T_5 \\ T_6 \end{bmatrix}_p \quad --(2)$$

β_0 denotes the parameter of the constant predictor, and T_t denotes time elapsed between baseline (time point 1) and time point t.

This enabled us to examine whether mindful awareness during meditation increased proportionally to the time elapsed since the baseline meditation session, assuming a linear proportional relationship between time elapsed and mindful awareness. The resulting regression slope ($\beta_{1,p}$) from each participant p was entered into a one-way Analysis of Variance (ANOVA) to examine group differences. This approach was followed independently for each SMS subscale, i.e., SMS-Mind and SMS-Body.

SM2.11 Offline fMRI data analysis

SM2.11.1 GLM activation analyses

Activation in the NF target ROI (PCC) during meditation relative to rest was estimated offline through GLM in FSL FEAT (https://web.mit.edu/fsl_v5.0.10/fsl/doc/wiki/FEAT.html; FSL v6.0.6.4). The canonical double-gamma hemodynamic response function (HRF) was convolved with the time course of each fMRI condition (meditation, rest, and NF, as shown in **Figure 1B**) in the NF runs to form the main condition predictors in the GLM. The remaining cue/instruction condition was implicitly modelled by the constant term in the GLM.

SM2.11.2 gPPI coupling analysis

The implementation of gPPI followed the GLM approach, with block design predictors in the first-level gPPI modelling the psychophysiological interactions between the seed region (PCC) and all voxels within CEN and SN. CEN and SN were outlined using the standard 7-network parcellation (Yeo et al., 2011), applied to a grey matter mask created by averaging all participant-level masks and thresholding at 0.3.

In the first-level GLM modelling for gPPI, the double-gamma HRF function was convolved with three block design time series: meditation minus rest, meditation plus rest, and NF condition, resulting in three condition predictors. The ‘meditation minus rest’ predictor captures the contrast of meditation > rest, isolating the differences between meditation and rest while excluding shared variance captured by the ‘meditation plus rest’ predictor. Although ‘meditation plus rest’ and ‘meditation minus rest’ span the same vector space as meditation and rest individually, the former approach directly isolates the desired contrast of meditation > rest. The predictors were zero-

centred and convolved with the mean PCC BOLD time series (seed time course) to form the PPI predictors. Overall, the first-level model included the three condition predictors, their respective PPI predictors and the seed time course as the non-nuisance regressors, thereby spanning the entire experimental space (McLaren et al., 2012).

---x---x---

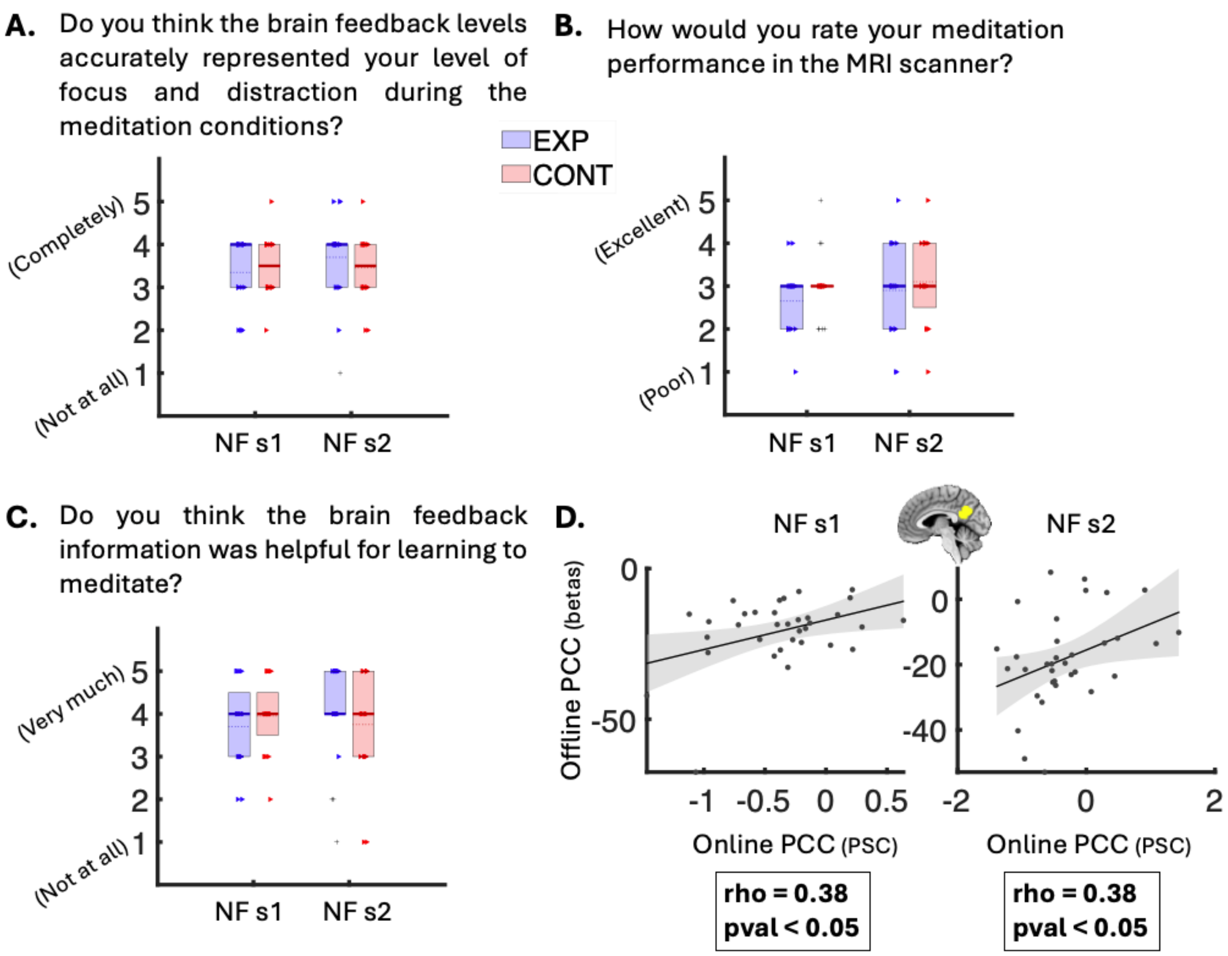

Figure S3:

Assessment of blinding effectiveness and the relationship between online and offline PCC signals. ***A) to C)*** *Box plots depicting participant ratings (1 to 5 scale) for each question indicated above the plots, collected at the end of each NF session. Blue represents the experimental group, and red represents the control group. No significant between-group differences in median ratings were observed for any of the questions (p > 0.05). Triangles indicate individual data points, with solid lines marking medians, dashed lines marking means, and pluses indicating outliers.* ***D)*** *Scatter*

plots displaying significant positive correlations between PCC activation betas denoised post-hoc (y-axis) and PCC activation PSC (x-axis) denoised during NF, for the contrast meditation vs. rest across the two NF sessions (s1: p = 0.028, rho = 0.38; s2: p = 0.028, rho = 0.38; N=34). Dots represent individual data points, with the shaded area showing the 95% confidence interval around the linear fit (solid black line). NF - neurofeedback; s1 - session 1; s2 - session 2; EXP - experimental group; CONT - control group; PCC - posterior cingulate cortex; PSC - percent signal change

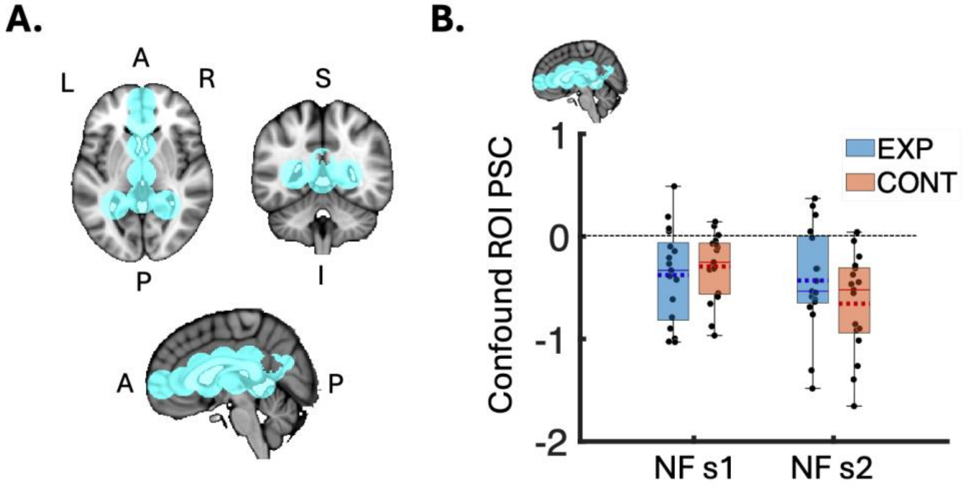

Figure S4:

*Control region of interest (ROI) used during NF as proxy for online denoising, and its percent signal change (PSC). **A)** Brain volume slices depicting the anatomical extent of the control ROI (in cyan) used as proxy for online physiological denoising during NF training. **B)** Box plot of mean PSC estimated online from the confound ROI during each NF session. There were no significant differences between groups in either session (p > 0.05; N(exp) = 17, N(cont) = 17). The box plot shows individual data points as black dots, outliers as triangles, data range as whiskers, means as dotted lines, and medians as solid lines. NF - neurofeedback; s1 - session 1; s2 - session 2; ROI - region of interest; PSC - percent signal change; EXP - experimental group; CONT - control group; P - posterior; L - left; R - right; I - inferior; S – Superior*

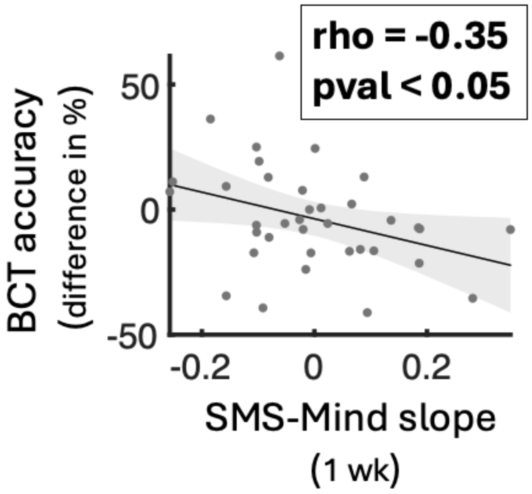

Figure S5:

Scatter plot of the post-hoc correlation analysis illustrating a significant negative correlation between increase in BCT accuracy from baseline to follow-up (y-axis; difference in BCT accuracy %) and decrease in slopes of mindful awareness of mental activity (x-axis; SMS-Mind slopes) (p = 0.044; rho = -0.35; N = 36). Individual data points are represented by dots, with the shaded area indicating the 95% confidence interval around the linear fit (solid black line). BCT - breath counting task; SMS - state mindfulness scale; wk - week

Table S2:

Summary of findings with key statistics

		**Cohen's *d***	**FDR-adjusted *p***

	Analysis summary	effect size OR correlation coefficient *r* (dfe)	value (Uncorrected *p* value)
**Behaviour**	5% FDR correction across all five behavioural tests below		
Change in emotional distress	ANCOVA group difference in total *DASS-21* difference scores	0.40 (34)	**0.041** (0.026)
Rate of change in mindful awareness of *mental activity* during real-world meditation	ANCOVA group difference in 1-week *SMS*-Mind slopes	0.41 (36)	**0.041** (0.019)
Rate of change in mindful awareness of *bodily sensations* during real-world meditation	ANCOVA group difference in 1-week *SMS*-Body slopes	0.02 (36)	0.888 (0.888)
Change in breath counting skill	ANCOVA group difference in *BCT* task performance accuracy	0.39 (32)	**0.041** (0.033)
	Wilcoxon rank sum test group difference in *BCT* probe accuracy	0.56 (34)	**0.005** (0.001)
**Brain**			
**Voxel-based**	5% FWE correction with 10,000 permutations		
Change in PCC-seeded negative coupling during NF meditation vs. rest	ANCOVA group difference from voxel-based gPPI analysis (NF session 1)	-	n.s.
	ANCOVA group difference from voxel-based gPPI analysis (NF session 2)	DLPFC cluster; 0.59 (29)	*FWE*-adjusted p = **0.032**; cluster size = 26 voxels
**ROI-based**	5% FDR correction across all six ROI-based tests below		
Change in PCC activation during NF meditation vs. rest	ANCOVA group difference (NF session 1)	0.04 (29)	0.876 (0.814)
	ANCOVA group difference (NF session 2)	0.03 (29)	0.876 (0.876)
Change in PCC-DLPFC FC from baseline to transfer meditation without NF (session 2 due to gPPI significance in session 2)	ANCOVA group difference in FC difference values	0.14 (28)	0.715 (0.477)
**Correlations**			
Correlation between PCC-DLPFC negative coupling (NF meditation session 2) and change in emotional distress	Pearson's partial correlation, controlling for age and gender	0.53 (28)	**0.018** (0.003)
Correlation between PCC-DLPFC negative coupling (NF meditation session 2) and slope of change in mindful awareness of mental activity	Pearson's partial correlation, controlling for age and gender	-0.14 (28)	0.715 (0.462)

Correlation between PCC-DLPFC negative coupling (NF meditation session 2) and change in breath counting accuracy	Pearson's partial correlation, controlling for age and gender	-0.23 (27)	0.660 (0.220)

FDR - Benjamini-Hochberg false discovery rate; FWE - family-wise error; n.s. - not significant; dfe - degrees of freedom; ANCOVA - analysis of covariance; DASS-21 - depression, stress, anxiety scale; SMS - state mindfulness scale; BCT - breath counting task; NF - neurofeedback; PCC - posterior cingulate cortex; DLPFC - dorsolateral prefrontal cortex; ROI - region of interest; gPPI - generalised psychophysiological interactions; FC - functional connectivity

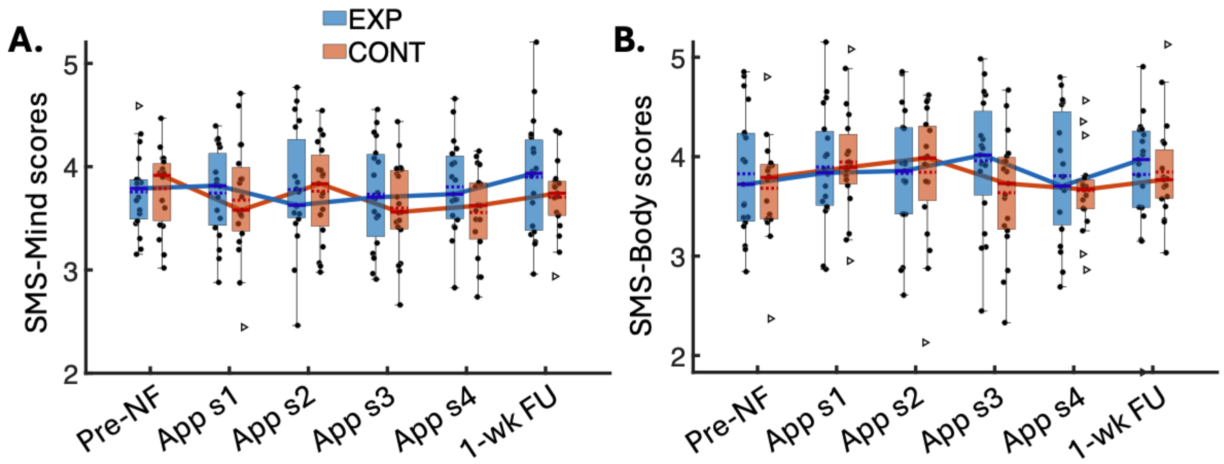

Figure S6:

Visualisation of changes in mindful awareness (SMS subscale scores) during 5-minute self-guided meditation sessions over one week post-NF. ***A)*** *Box plot showing changes in mindful awareness of mental activity (SMS-Mind) over time in each group, adjusted for pre-meditation SMS-Mind, sleepiness, age, and sex.* ***B)*** *Similar plot for mindful awareness of bodily sensations (SMS-Body). Dots represent individual scores, triangles denote outliers, whiskers indicate data range, and the solid and dashed lines within each box represent the median and mean, respectively. Medians across time points are connected to illustrate trends, with blue for the experimental group and red for the control group. NF - neurofeedback; sx - session x; EXP - experimental group; CONT - control group; FU - follow up; SMS - state mindfulness scale; wk – week*

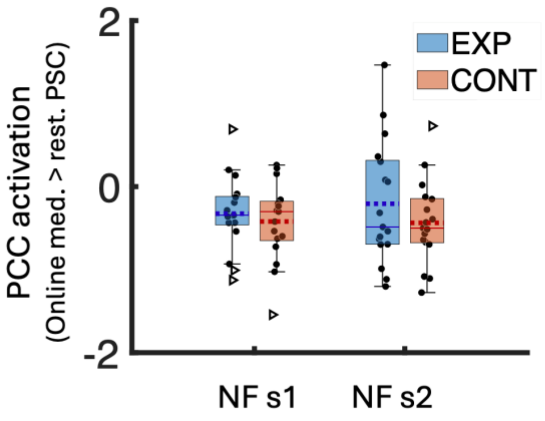

Figure S7:

Between-group differences in online-estimated mean PCC activation. Box plot of group difference (not significant) in mean PCC activation percent signal change (PSC) calculated online (online-denoised) during NF-guided meditation vs. rest in each NF session (N(exp) = 17, N(cont) = 17). Y-axis represents mean PCC PSC. These observations are consistent with the offline estimates presented in Figure 4B.

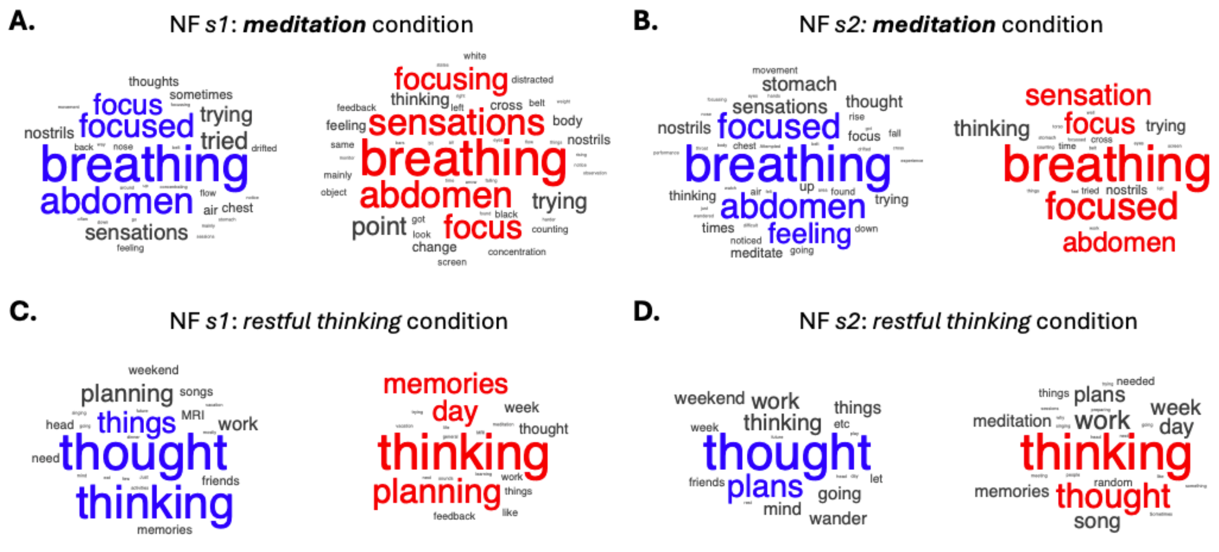

Figure S8:

Word cloud visualization of mental strategies used by each group (N(exp) = 19; N(cont) = 19) during meditation and restful thinking conditions across NF sessions. A) Terms organized by

frequency from participants' self-reported strategies during meditation in NF session 1: cloud with blue terms for the experimental group (left), cloud with red terms for the control group (right). Word size reflects frequency, with the most frequently used words highlighted in color. B) Same as (A) for NF session 2. C) Same as (A) for restful thinking in session 1. D) Same as (C) for session 2. NF - neurofeedback; s1 - session 1; s2 - session 2.

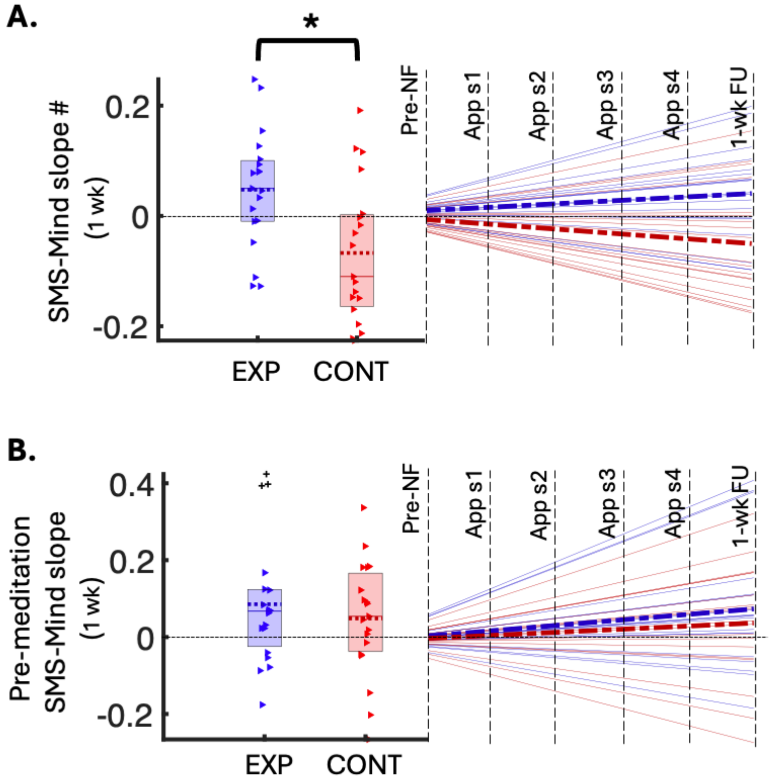

Figure S9:

Sensitivity analyses considering 1-week changes in pre-meditation mindful awareness. A) Left: Box plot of group differences in slopes of change (y-axis) in SMS-Mind scores over 1-week meditation practice (5-minute sessions), after controlling for age, sex, arousal, but not pre-meditation mindful awareness (uncorrected p = 0.005, Cohen's d = 0.50, N(exp) = 19, N(cont) = 19). Right: Same data represented using a line graph, with the pre-NF data normalised to 0, group mean slopes indicated as bold dashed lines and individual slopes shown as faded solid lines (experimental in blue, control in red). B) Same as (A), but for slopes of change (y-axis) in pre-meditation SMS-Mind scores over 1-week, after controlling for age, sex, and arousal (not

*significant, uncorrected p>0.05, (N(exp) = 19, N(cont) = 19)). EXP - Experimental group; CONT - Control group; SMS - State Mindfulness Scale; App sx - App meditation session x; wk - week; FU - Follow up; * uncorrected-significant p<0.05; # pre-meditation SMS-Mind not a covariate*

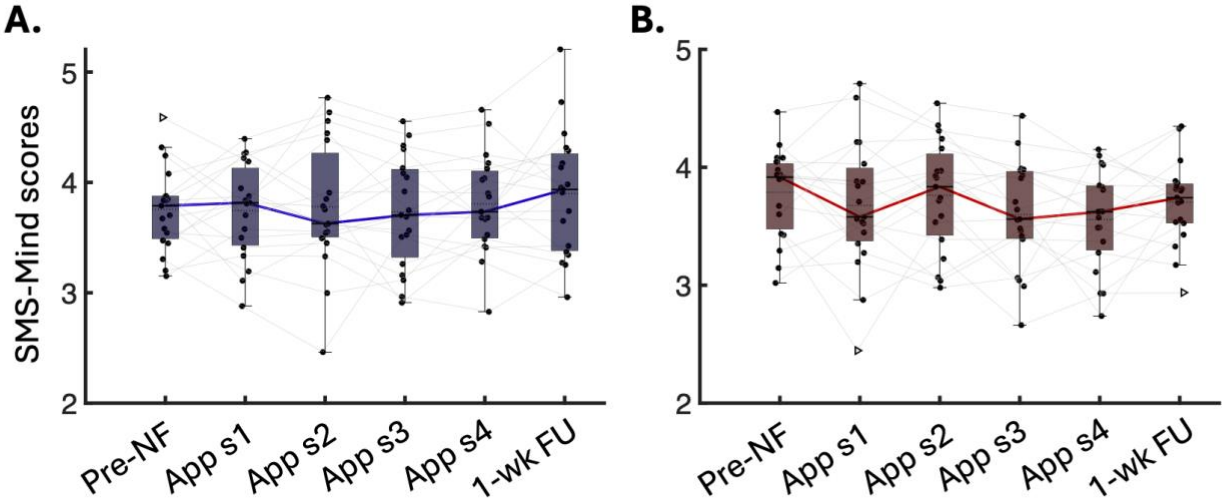

Figure S10:

Within-group changes in mindful awareness (SMS-Mind) over 1 week of self-guided practice. A) Box plot of post-meditation SMS-Mind scores (y-axis) across time points (x-axis) over the 1-week meditation practice (5-minute sessions) in the experimental group, adjusted for age, sex, arousal, and pre-meditation SMS-Mind (N(exp) = 19). Each participant's SMS-Mind score at each timepoint is connected by a faded line, and the darker blue line connects the median scores across timepoints. B) Same as (A), but for the control group (N(cont)=19), with the darker red line connecting the median scores across timepoints. SMS - State Mindfulness Scale; App sx - App meditation session x; wk - week; FU - Follow up

References

Andersson, J. L. R., Skare, S., & Ashburner, J. (2003). How to correct susceptibility distortions in spin-echo echo-planar images: application to diffusion tensor imaging. NeuroImage, 20(2), 870–888.

Arch, J. J., & Craske, M. G. (2006). Mechanisms of mindfulness: Emotion regulation following a focused breathing induction. Behaviour Research and Therapy, 44(12), 1849–1858.

Avants, B. B., Tustison, N., Song, G., & Others. (2009). Advanced normalization tools (ANTS). The Insight Journal, 2(365), 1–35.

Baer, R. A., Smith, G. T., Hopkins, J., Krietemeyer, J., & Toney, L. (2006). Using self-report assessment methods to explore facets of mindfulness. Assessment, 13(1), 27–45.

Barnes, L. L. B., Harp, D., & Jung, W. S. (2002). Reliability Generalization of Scores on the Spielberger State-Trait Anxiety Inventory. Educational and Psychological Measurement, 62(4), 603–618.

Behzadi, Y., Restom, K., Liau, J., & Liu, T. T. (2007). A component based noise correction method (CompCor) for BOLD and perfusion based fMRI. NeuroImage, 37(1), 90–101.

Britton, W. B., Lindahl, J. R., Cahn, B. R., Davis, J. H., & Goldman, R. E. (2014). Awakening is not a metaphor: the effects of Buddhist meditation practices on basic wakefulness. Annals of the New York Academy of Sciences, 1307, 64–81.

Buysse, D. J., Reynolds, C. F., 3rd, Monk, T. H., Berman, S. R., & Kupfer, D. J. (1989). The Pittsburgh Sleep Quality Index: a new instrument for psychiatric practice and research. Psychiatry Research, 28(2), 193–213.

Christopher, M. S., Neuser, N. J., Michael, P. G., & Baitmangalkar, A. (2012). Exploring the Psychometric Properties of the Five Facet Mindfulness Questionnaire. Mindfulness, 3(2), 124–131.

Esteban, O., Markiewicz, C. J., Blair, R. W., Moodie, C. A., Isik, A. I., Erramuzpe, A., Kent, J. D., Goncalves, M., DuPre, E., Snyder, M., Oya, H., Ghosh, S. S., Wright, J., Durnez, J., Poldrack, R. A., & Gorgolewski, K. J. (2019). fMRIPrep: a robust preprocessing pipeline for functional MRI. Nature Methods, 16(1), 111–116.

Fino, E., Martoni, M., & Russo, P. M. (2021). Specific mindfulness traits protect against negative effects of trait anxiety on medical student wellbeing during high-pressure periods. Advances in Health Sciences Education: Theory and Practice, 26(3), 1095–1111.

Ganesan, S., A. Moffat, B., Van Dam, N. T., Lorenzetti, V., & Zalesky, A. (2023). Meditation attenuates default-mode activity: A pilot study using ultra-high field 7 tesla MRI. Brain Research Bulletin, 203, 110766.

Ganesan, S., Beyer, E., Moffat, B., Van Dam, N. T., Lorenzetti, V., & Zalesky, A. (2022). Focused attention meditation in healthy adults: A systematic review and meta-analysis of cross-sectional functional MRI studies. Neuroscience and Biobehavioral Reviews, 141, 104846.

Greve, D. N., & Fischl, B. (2009). Accurate and robust brain image alignment using boundary-based registration. NeuroImage, 48(1), 63–72.

Henry, J. D., & Crawford, J. R. (2005). The short-form version of the Depression Anxiety Stress Scales (DASS-21): construct validity and normative data in a large non-clinical sample. The British Journal of Clinical Psychology / the British Psychological Society, 44(Pt 2), 227–239.

Hoddes, E., Zarcone, V., Smythe, H., Phillips, R., & Dement, W. C. (1973). Quantification of sleepiness: a new approach. Psychophysiology, 10(4), 431–436.

Jenkinson, M., Bannister, P., Brady, M., & Smith, S. (2002). Improved optimization for the robust and accurate linear registration and motion correction of brain images. NeuroImage, 17(2), 825–841.

Kasper, L., Bollmann, S., Diaconescu, A. O., Hutton, C., Heinzle, J., Iglesias, S., Hauser, T. U., Sebold, M., Manjaly, Z.-M., Pruessmann, K. P., & Stephan, K. E. (2017). The PhysIO Toolbox for Modeling Physiological Noise in fMRI Data. Journal of Neuroscience Methods, 276, 56–72.

Khoury, B., Sharma, M., Rush, S. E., & Fournier, C. (2015). Mindfulness-based stress reduction for healthy individuals: A meta-analysis. Journal of Psychosomatic Research, 78(6), 519–528.

Levinson, D. B., Stoll, E. L., Kindy, S. D., Merry, H. L., & Davidson, R. J. (2014). A mind you can count on: validating breath counting as a behavioral measure of mindfulness. Frontiers in Psychology, 5, 1202.

Lovibond, S. H., & Lovibond, P. F. (1996). Manual for the Depression Anxiety Stress Scales.

McLaren, D. G., Ries, M. L., Xu, G., & Johnson, S. C. (2012). A generalized form of context-dependent psychophysiological interactions (gPPI): a comparison to standard approaches. NeuroImage, 61(4), 1277–1286.

Mollayeva, T., Thurairajah, P., Burton, K., Mollayeva, S., Shapiro, C. M., & Colantonio, A. (2016). The Pittsburgh sleep quality index as a screening tool for sleep dysfunction in clinical and non-clinical samples: A systematic review and meta-analysis. Sleep Medicine Reviews, 25, 52–73.

Mrazek, M. D., Phillips, D. T., Franklin, M. S., Broadway, J. M., & Schooler, J. W. (2013). Young and restless: validation of the Mind-Wandering Questionnaire (MWQ) reveals disruptive impact of mind-wandering for youth. Frontiers in Psychology, 4, 560.

Reuter, M., Schmansky, N. J., Rosas, H. D., & Fischl, B. (2012). Within-subject template estimation for unbiased longitudinal image analysis. NeuroImage, 61(4), 1402–1418.

Ros, T., Enriquez-Geppert, S., Zotev, V., Young, K. D., Wood, G., Whitfield-Gabrieli, S., Wan, F., Vuilleumier, P., Vialatte, F., Van De Ville, D., Todder, D., Surmeli, T., Sulzer, J. S., Strehl, U., Sterman, M. B., Steiner, N. J., Sorger, B., Soekadar, S. R., Sitaram, R., … Thibault, R. T. (2020). Consensus on the reporting and experimental design of clinical and cognitive-behavioural neurofeedback studies (CRED-nf checklist). Brain: A Journal of Neurology, 143(6), 1674–1685.

Ruimi, L., Hadash, Y., Tanay, G., & Bernstein, A. (2022). State Mindfulness Scale (SMS). In O. N. Medvedev, C. U. Krägeloh, R. J. Siegert, & N. N. Singh (Eds.), Handbook of Assessment in Mindfulness Research (pp. 1–16). Springer International Publishing.

Smith, S. M., Jenkinson, M., Woolrich, M. W., Beckmann, C. F., Behrens, T. E. J., Johansen-Berg, H., Bannister, P. R., De Luca, M., Drobnjak, I., Flitney, D. E., Niazy, R. K., Saunders, J., Vickers, J., Zhang, Y., De Stefano, N., Brady, J. M., & Matthews, P. M. (2004). Advances in functional and structural MR image analysis and implementation as FSL. NeuroImage, 23 Suppl 1, S208–S219.

Spielberger, C. D., Gonzalez-Reigosa, F., Martinez-Urrutia, A., Natalicio, L. F. S., & Natalicio, D. S. (1971). The State-Trait Anxiety Inventory. Revista Interamericana de

Psicología/Interamerican Journal of Psychology, 5(3 & 4). https://doi.org/10.30849/rip/ijp.v5i3 & 4.620
Tanay, G., & Bernstein, A. (2013). State Mindfulness Scale (SMS): development and initial validation. Psychological Assessment, 25(4), 1286–1299.
Yeo, B. T. T., Krienen, F. M., Sepulcre, J., Sabuncu, M. R., Lashkari, D., Hollinshead, M., Roffman, J. L., Smoller, J. W., Zöllei, L., Polimeni, J. R., Fischl, B., Liu, H., & Buckner, R. L. (2011). The organization of the human cerebral cortex estimated by intrinsic functional connectivity. Journal of Neurophysiology, 106(3), 1125–1165.
